# EARDC: Energy-Aware Reaction–Diffusion-Based Clustering for Area Coverage in Wireless Sensor Networks

**DOI:** 10.3390/s26175505

**Published:** 2026-08-30

**Authors:** Shu-Yuan Wu, Theodore Brown

**Affiliations:** 1 Graduate Center, City University of New York, New York, NY 10016, USA; tbrown@gc.cuny.edu; 2 Queens College, City University of New York, Queens, NY 11367, USA

**Keywords:** wireless sensor networks (WSNs), clustering protocol, energy-efficient, reaction–diffusion, self-organizing, location-unaware, coordinate-free, area coverage, network lifetime

## Abstract

Energy-efficient clustering extends the lifetime of wireless sensor networks (WSNs). Conventional protocols such as LEACH and HEED primarily address communication energy and cluster head (CH) selection rather than area coverage under random deployment. C3 considers coverage and redundancy without node coordinates; however, its virtual-ring reconfiguration is initiated by a sink or reference node. In this paper, we propose Energy-Aware Reaction–Diffusion Clustering (EARDC), a self-organizing, coordinate-free, and decentralized framework for energy-efficient area coverage in randomly deployed WSNs. EARDC determines CH formation, cluster membership, and temporary sleep states from information exchanged among one-hop neighbors. It requires neither exact node coordinates, sink-initiated reconfiguration, nor network-wide coverage information computed at the base station. A residual-energy-scaled activator–inhibitor update supports self-organized CH selection, and local membership control determines the active sensing set from selected CHs and accepted members. Other non-CH nodes sleep until the next scheduled clustering epoch. Analytical approximations for active-node density and coverage are evaluated against run-level simulation measurements to assess their accuracy and limitations. Simulations at three deployment densities characterize the tradeoff among network lifetime, coverage retention, active participation, and sensing redundancy. EARDC provides longer network lifetime and a smaller active fraction than LEACH, HEED, and WIFN. C3 provides stronger redundancy suppression. In the dense deployment, the lifetime-maximizing EARDC setting has shorter coverage retention than C3 but a longer coverage-bounded lifetime. These results show that network lifetime and sustained area coverage represent distinct performance objectives.

## 1. Introduction

Wireless sensor networks (WSNs) are widely used for environmental monitoring, infrastructure monitoring, disaster management, and cyber-physical applications [1]. A typical WSN consists of distributed sensor nodes with sensing, computation, and communication capabilities. These nodes monitor an area of interest and transmit sensed data to designated sinks or base stations. In remote or hostile environments, sensor nodes are often battery-powered and may be difficult to replace or recharge. Prolonging network lifetime while preserving sensing coverage is therefore a fundamental WSN design objective.

Balancing sensing coverage and energy consumption remains challenging. A larger active sensing set can improve coverage and fault tolerance, although it may also accelerate energy depletion. Reducing active participation conserves energy at the risk of lower area coverage. Existing approaches have made substantial progress in communication energy reduction and sensing coverage preservation [2,3,4,5]. Many coverage-preserving scheduling and optimization models rely on centralized algorithms, deterministic deployment, exact node coordinates, or base-station knowledge of node positions, sensing disks, coverage holes, and overlap regions. Such information supports optimization and analysis. However, collecting and maintaining network-wide location and coverage information can impose substantial communication and coordination overhead in large random deployments. Random deployment methods such as aerial scattering can also result in uneven node spacing and an unpredictable network topology. These conditions motivate decentralized mechanisms that maintain sensing coverage and energy efficiency using only local information.

Clustering is widely used to reduce communication cost and distribute energy consumption by organizing sensor nodes into groups [4,6]. In a clustered WSN, selected cluster heads (CHs) receive data from member nodes, aggregate the data, and transmit the aggregated data to a sink or base station. Low-Energy Adaptive Clustering Hierarchy (LEACH) uses probabilistic CH rotation [7], and Hybrid Energy-Efficient Distributed clustering (HEED) uses residual energy and intra-cluster communication cost in CH election [8]. These protocols primarily address communication energy and CH selection; active sensing control for area coverage is not their primary design objective.

Reaction–diffusion (RD) systems provide a basis for decentralized pattern formation. Originally introduced to explain biological pattern formation [9], RD models generate self-organized patterns through local reaction and diffusion processes. In WSNs, these local interactions can support distributed CH selection without centralized coordination or predefined geometric partitions. This property makes RD suitable for random deployments with irregular node placement.

In this work, we develop an energy-aware RD clustering framework for area coverage in randomly deployed WSNs. Normalized residual energy is integrated into the local activator–inhibitor updates used for CH selection. Cluster membership and temporary sleep-state control determine which non-CH nodes are included in the active sensing set. Each non-CH node uses locally estimated distances and predefined thresholds to determine whether it lies in the inner or outer region of each advertised CH within communication range. Because CHs also perform sensing, nodes in the inner region have greater overlap with the CH sensing disk. Their join-request probabilities are reduced, and their requests receive lower priority during CH acceptance to limit redundant participation. Nodes in the outer region of a CH have less sensing overlap with the CH. Their join-request probabilities are generally higher, and their requests receive priority during CH acceptance. These decisions require neither exact node coordinates nor a centralized coverage map. In each round, selected CHs and accepted members perform sensing. Members transmit sensed data to their CHs, and CHs aggregate the received data and transmit them to the base station.

EARDC is a decentralized, coordinate-free, and self-organizing framework. It does not require exact node coordinates, a centralized coverage map, or clustering and coverage decisions computed at the base station. CH selection and cluster membership use information from one-hop neighbors and local range estimates derived from received signal strength indicator (RSSI) or similar link-level measurements. Coverage preservation and redundancy suppression are therefore addressed through local cluster formation and sleep-state control rather than through a separate centralized activation process.

Maintaining area coverage and conserving energy without exact node coordinates or centralized coverage computation remain challenging in randomly deployed WSNs. This study examines the tradeoff produced by residual-energy-aware CH selection and local sensing participation control. The main contributions are summarized as follows:

- An energy-aware RD CH-selection mechanism is introduced. Normalized residual energy scales the local activator–inhibitor update before CHs are selected from local activator maxima.
- A coordinate-free membership and temporary sleep-state control procedure is coupled with RD-based CH selection. Selected CHs and accepted members form the active sensing set instead of all alive nodes.
- Analytical approximations describe the relationships among CH probability, conditional non-CH participation, active-node density, coverage probability, overlap, and redundancy. Their accuracy and limitations are evaluated through quantitative comparison with simulation results.
- Round-based simulations under a common radio model compare EARDC with LEACH, HEED, C3, and WIFN. The evaluation characterizes tradeoffs among network lifetime, first node death (FND), coverage retention, coverage-bounded lifetime, active ratio, and redundancy. Statistical tests assess protocol-level differences, and an ablation study isolates the contribution of membership and sensing participation control.

The remainder of this paper is organized as follows. Section 2 reviews coverage control, clustering protocols, bio-inspired optimization, and RD models in WSNs. Section 3 presents the EARDC system model and protocol operations, including RD-based CH selection, cluster membership, and sleep-state control. Section 4 analyzes the homogeneous equilibrium, linear stability, and characteristic wavelength of the energy-aware RD update. Section 5 presents a CH-separation reference and aggregate expressions for active-node density, coverage, and overlap under hexagonal and random deployment models. Section 6 presents the simulation setup and comparative results. Section 7 concludes the paper and outlines future research directions.

## 2. Related Work

Research on WSN coverage and clustering includes several related areas. Coverage studies define sensing objectives, coverage metrics, and node scheduling strategies. Clustering protocols address CH selection, data aggregation, and energy balancing. Bio-inspired and optimization-based methods support CH selection, routing, and deployment planning. RD methods use local interactions for distributed topology formation and clustering. This section positions EARDC with respect to existing coverage-aware, energy-aware, and self-organizing WSN mechanisms.

### 2.1. Coverage in WSNs

Coverage in WSNs is commonly categorized as point, area, or barrier coverage [10]. Point coverage monitors predefined target locations. Area coverage monitors an entire region or a required portion of it. Barrier coverage detects movement across a monitored path or boundary. These coverage objectives lead to different deployment and scheduling requirements.

WSN deployment is commonly classified as deterministic or random according to the degree of control over node placement. Deterministic grid or hexagonal layouts provide predictable coverage. Such placement may be impractical in inaccessible or hazardous environments. Random deployment, often modeled using stochastic spatial processes, reflects uncontrolled placement methods such as aerial scattering. It can result in an uneven node distribution and nonuniform coverage [11,12,13].

Many coverage control and scheduling models assume access to global topology information, node coordinates, or a centralized coverage map. Such information enables geometric optimization of the active sensing set, coverage gaps, and overlap. It also requires the collection and maintenance of network-wide location and coverage information. Coordinate-free methods instead determine node participation without exact node positions.

Sensor activity scheduling addresses the energy–coverage tradeoff by controlling which sensors remain active. Redundant nodes can enter sleep states to reduce energy consumption and preserve useful sensing coverage [2,14]. In many protocols, activity scheduling is performed after cluster formation and independently of CH selection. The active sensing set and CH roles are therefore determined by separate mechanisms, even though both affect coverage and energy consumption.

Recent coverage studies apply learning-based, probabilistic, and swarm-intelligence methods. Deep reinforcement learning (DRL) and graph neural networks (GNNs) have been used for adaptive sensor configuration [15]. Stochastic geometry has been applied to coverage–energy tradeoffs [16], and topology-based machine learning has been used for coverage-hole detection [17]. Yang et al. [18] use an improved Cuckoo Search algorithm to adjust node positions and increase grid-based coverage. These studies address coverage analysis, deployment optimization, and active sensor configuration. Their information requirements vary; several methods use explicit deployment geometry or network-wide state.

### 2.2. Clustering Algorithms

Clustering protocols reduce communication overhead by assigning data aggregation and long-distance transmission to selected CHs. LEACH introduced randomized CH rotation to distribute the CH communication load among nodes over time [7,19,20,21,22]. Its CH election is simple and distributed. It does not consider sensing coverage, residual energy, or local redundancy in CH selection. Consequently, CH locations may be uneven under random deployment. Under LEACH, alive nodes generally continue sensing because the protocol does not include coverage-oriented sleep-state control.

HEED uses residual energy as its primary CH election parameter and intra-cluster communication cost as a secondary parameter [8]. Related variants, including H-HEED and EHEED, address heterogeneous node energy and inter-cluster routing load [23,24]. In these protocols, residual energy enters the CH election rule directly. In EARDC, normalized residual energy instead scales the activator–inhibitor update used for CH selection.

Threshold-sensitive Energy Efficient sensor Network (TEEN) and Adaptive Periodic TEEN (APTEEN) use hard and soft thresholds to reduce data transmission under event-driven and hybrid reporting [25,26,27]. These thresholds control when nodes report sensed data rather than which nodes remain active for area coverage. The protocols do not primarily address sensing redundancy or residual-energy-aware CH placement.

Some protocols combine clustering and coverage control. The Coverage, Connectivity, and Communication (C3) protocol uses RSSI-derived virtual rings and redundancy detection for clustering and sleep scheduling [28]. C3 is coordinate-free; however, virtual ring reconfiguration is initiated by a sink (base station) or reference node. EARDC uses a different mechanism. Nodes exchange activator and inhibitor values locally, and normalized residual energy scales the update used for CH selection. Cluster membership decisions then determine which non-CH nodes remain active or enter a temporary sleep state until the next scheduled clustering epoch.

Surveys and comparative studies have documented the strengths and limitations of clustering and communication protocols under different deployment settings [3,6,21,22,29,30]. Clustering studies commonly emphasize communication energy and network lifetime, and coverage studies focus on active sensor selection. For area coverage, CH roles and the active sensing set must be considered together because both affect sensing coverage, energy consumption, and subsequent node availability.

### 2.3. Bio-Inspired and Optimization-Based Clustering

Recent WSN clustering studies use machine learning, fuzzy logic, evolutionary search, and bio-inspired optimization for CH selection and routing [4,31]. Swarm-intelligence methods such as Particle Swarm Optimization (PSO), Ant Colony Optimization (ACO), and Artificial Bee Colony (ABC) use population-based search to optimize CH selection, routing, or cluster stability [32,33,34,35,36,37,38]. Evolutionary methods such as Genetic Algorithms (GAs) and Differential Evolution (DE) use fitness functions that may include energy, coverage quality, and network lifetime [39]. These methods support multiple objectives, although their iterative searches can add computation and coordination overhead.

Energy-aware metaheuristic protocols include additional energy and routing criteria. SHOE combines swarm-based optimization with nonuniform clustering and multi-hop routing based on residual energy, communication distance, base-station distance, and energy variance [40]. Hybrid Energy Harvesting Optimization with Cultural Algorithm-Optimal Cluster Head Selection (HEHO-CA-OCHS) uses population-based search and a multi-criteria fitness function based on residual energy, distance, and delay [41]. These methods use explicit search to select CHs or routing paths. EARDC instead selects CHs from activator values updated through local node exchanges without population-based search.

### 2.4. Reaction–Diffusion Models in WSNs

RD models provide another approach to distributed CH selection. Instead of using independent CH probabilities or explicit optimization, nodes update activator and inhibitor values through local exchanges, and local activator maxima are used for CH selection. EARDC extends this approach by integrating normalized residual energy into the update and combining CH selection with local sensing participation.

Wakamiya et al. [42] developed an RD-based topology control method for periodic data gathering. Sensors formed clusters and multi-hop data-gathering paths through local activator–inhibitor interactions. The reported results showed lower energy consumption and data collection delay than direct or single-hop transmission. The method used RD for distributed routing and cluster formation but did not control sensing participation for area coverage.

Other RD studies focus on communication paths rather than sensing participation. Lowe and Miorandi [43] proposed a distributed method for establishing data highways in dense sensor networks. Local interactions and diffusion filtering formed elongated regions of highly activated nodes that supported high-throughput forwarding. Miorandi et al. [44] extended that method to networks with incomplete knowledge of sink locations. These studies use RD for routing and data forwarding rather than selecting active sensors for area coverage.

RD models have also been applied to CH election. Yamamoto and Miorandi [45,46] evaluated the Gierer–Meinhardt and Activator–Substrate models for fully distributed CH election. Nodes at local activator maxima were elected as CHs, and the reported results showed stable cluster formation. Wu et al. [47] combined the Gierer–Meinhardt model with decentralized intra-cluster activation control, in which local state transitions determined member activity after RD-based clustering. These studies are closely related to EARDC. Unlike EARDC, they do not integrate normalized residual energy into the update used for CH selection and active sensing control.

RD behavior under irregular node placement and implementation constraints has also been studied. Henderson et al. [48,49] demonstrated that Turing-type RD models could form stable patterns under irregular sensor distributions and structural perturbations. Hyodo et al. [50,51] reported rapid convergence in grid-based sensor networks through simulations and physical prototypes. These studies support the feasibility of distributed RD updates in sensor networks.

These studies establish RD as a distributed method for topology formation, routing, and clustering [52]. EARDC differs by integrating normalized residual energy into the activator–inhibitor update and combining RD-based CH selection with local control of sensing participation.

### 2.5. Summary

Table 1 summarizes the reviewed WSN coverage and clustering methods. Coverage studies address sensing criteria and active/sleep scheduling. Conventional clustering protocols reduce communication cost and balance CH energy, and C3 coordinates clustering with sleep-state control. Learning-based and metaheuristic methods use optimization for CH selection, routing, or coverage. RD methods use local node interactions for distributed topology formation and clustering. EARDC differs from the reviewed methods by integrating normalized residual energy into the RD update used for CH selection and determining the active sensing set through local membership and sleep-state control. It requires neither exact node coordinates nor a centralized coverage map.

## 3. Energy-Aware Decentralized Clustering and Coverage Model

**Figure 1:**
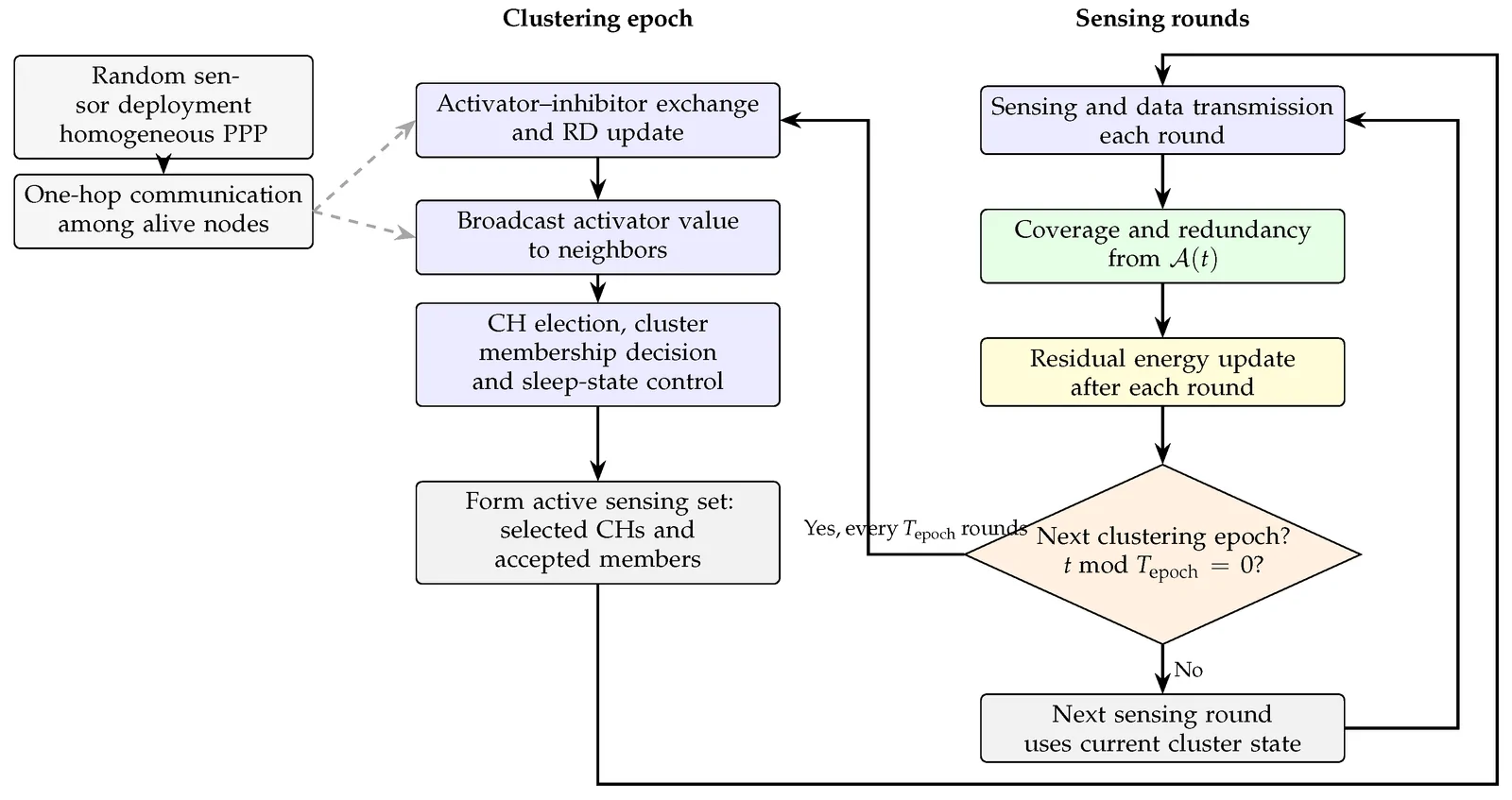
High-level EARDC operation during sensing rounds and clustering epochs. Solid arrows indicate protocol flow, and dashed arrows identify operations that use one-hop communication.

This section presents EARDC, a self-organizing, coordinate-free, and decentralized framework for cluster formation and sensing participation. EARDC distinguishes between sensing rounds and clustering epochs. A round consists of sensing, data transmission, and residual energy updates. A clustering epoch is a scheduled reclustering step performed once every $T_{\mathrm{epoch}}$ rounds. Active nodes sense and participate in data transmission in every round; EARDC does not use event-triggered reporting. Activator and inhibitor values are exchanged and updated only at clustering epochs. Figure 1 summarizes the round-level and clustering-epoch operations.

Initially, sensors are randomly deployed in the monitoring field according to a homogeneous Poisson point process (PPP). At the beginning of each clustering epoch, sensors exchange activator and inhibitor values with one-hop neighbors, update their activator–inhibitor values, broadcast current activator values to neighbors, and compare activator values of neighbors within the CH separation radius $R_{\mathrm{CH}}$. Nodes with local activator maxima announce the CH role to one-hop neighbors. Non-CH nodes calculate join probabilities to determine whether to send a join request to a CH, and CHs accept or reject requests based on local distance regions. Selected CHs and accepted members form the active sensing set; all other non-CH nodes enter a temporary sleep state until the next clustering epoch. The active sensing set remains unchanged until the next scheduled clustering epoch, except that nodes with exhausted energy are excluded. In each round, selected CHs and accepted members perform sensing. Members transmit sensed data to their CHs, and CHs aggregate and forward the data to the base station.

Normalized residual energy scales the Gierer–Meinhardt activator–inhibitor update used for CH selection [53]. Local membership and sleep-state control determine the active sensing set and limit simultaneous sensing in overlapping coverage areas. Selected CHs and accepted members consume energy through sensing and data transmission in each round. Their updated residual energy is used at the next clustering epoch, when EARDC updates the activator and inhibitor values and selects new CH and member roles.

EARDC distinguishes among alive, sleeping, and active nodes. An alive node has residual energy above the operational energy threshold. A sleeping node is alive but does not sense, transmit or receive data, exchange control messages, or participate in CH selection and cluster membership. After join requests are processed at the beginning of a clustering epoch, rejected or unclustered non-CH nodes sleep until the next scheduled clustering epoch. An active node is an alive, non-sleeping node selected as a CH or accepted as a cluster member. Active nodes form the active sensing set A(t) and perform sensing and data transmission in each round.

### 3.1. Sensor Deployment

WSN deployment may be deterministic, random, or hybrid, depending on the application and the degree of placement control. Deterministic deployment uses planned sensor locations, such as grid or hexagonal layouts. Random deployment places sensors without precise location control and is applicable to unattended or hazardous environments. Hybrid deployment combines planned and random placement in different parts of the monitoring region.

This work considers random deployment of stationary sensors. The initial sensor locations are modeled by a homogeneous PPP with density λ over the monitoring region. The parameter λ denotes the average number of sensors per unit area. EARDC uses local range estimates rather than exact node coordinates. CH roles, cluster memberships, sleep states, and residual energies evolve over time.

The homogeneous PPP models independent random sensor placement. Consider a two-dimensional monitoring region $A_{\mathrm{total}}$, and let N(A) denote the number of sensors in a measurable subregion $A\subseteq A_{\mathrm{total}}$. For density λ, N(A) follows a Poisson distribution with mean λ|A|, where |A| is the area of *A*. The probability of observing exactly *n* sensors in *A* is

(1) $$P\left\{N\left(A\right)=n\right\}=\frac{e^{-\lambda |A|}{\left(\lambda |A|\right)}^{n}}{n!},\;n=0,1,2,\ldots .$$

For a homogeneous PPP, sensor counts in mutually disjoint regions are independent. For measurable disjoint subregions $A_{1},A_{2},\ldots ,A_{k}\subseteq A_{\mathrm{total}}$, this property is expressed as

(2) $$P\left(N\left(A_{1}\right)=n_{1},\;N\left(A_{2}\right)=n_{2},\;\ldots ,\;N\left(A_{k}\right)=n_{k}\right)=\prod_{i=1}^{k}P\left\{N\left(A_{i}\right)=n_{i}\right\},$$

where $N(A_{i})$ is the number of sensors in $A_{i}$, and $n_{i}$ is a nonnegative integer.

### 3.2. Reaction–Diffusion-Based Cluster Head Selection and Cluster Formation

**Figure 2:**
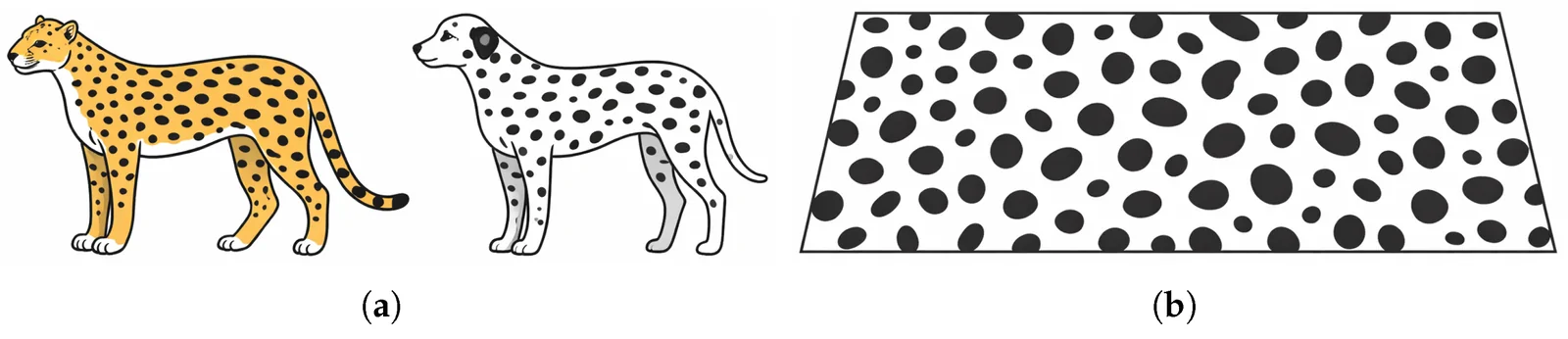
Conceptual illustrations of reaction–diffusion-like spot patterns generated using an OpenAI image model. (**a**) Spotted animal coats as qualitative examples of biological patterning. (**b**) An idealized two-dimensional spot pattern. The images are included only for visualization and are not used as simulation data or analytical evidence.

Reaction–diffusion models were introduced by Turing [9] to explain pattern formation through local reaction and diffusion processes. EARDC applies this principle to distributed CH selection. Sensors exchange activator and inhibitor values with one-hop neighbors, and alive nodes with local activator maxima within $R_{\mathrm{CH}}$ are selected as CHs. Figure 2 provides a conceptual illustration of reaction–diffusion-like spot formation.

Activator–inhibitor models are a widely used class of reaction–diffusion models. They use two interacting values with opposing effects. The activator reinforces local growth and drives inhibitor production, and the inhibitor limits activator growth. EARDC uses these interactions for distributed CH selection. Each sensor maintains activator and inhibitor values, and local activator maxima are used to select CHs. The inhibitor term suppresses activator growth around a local maximum, which limits nearby competing maxima.

**Figure 3:**
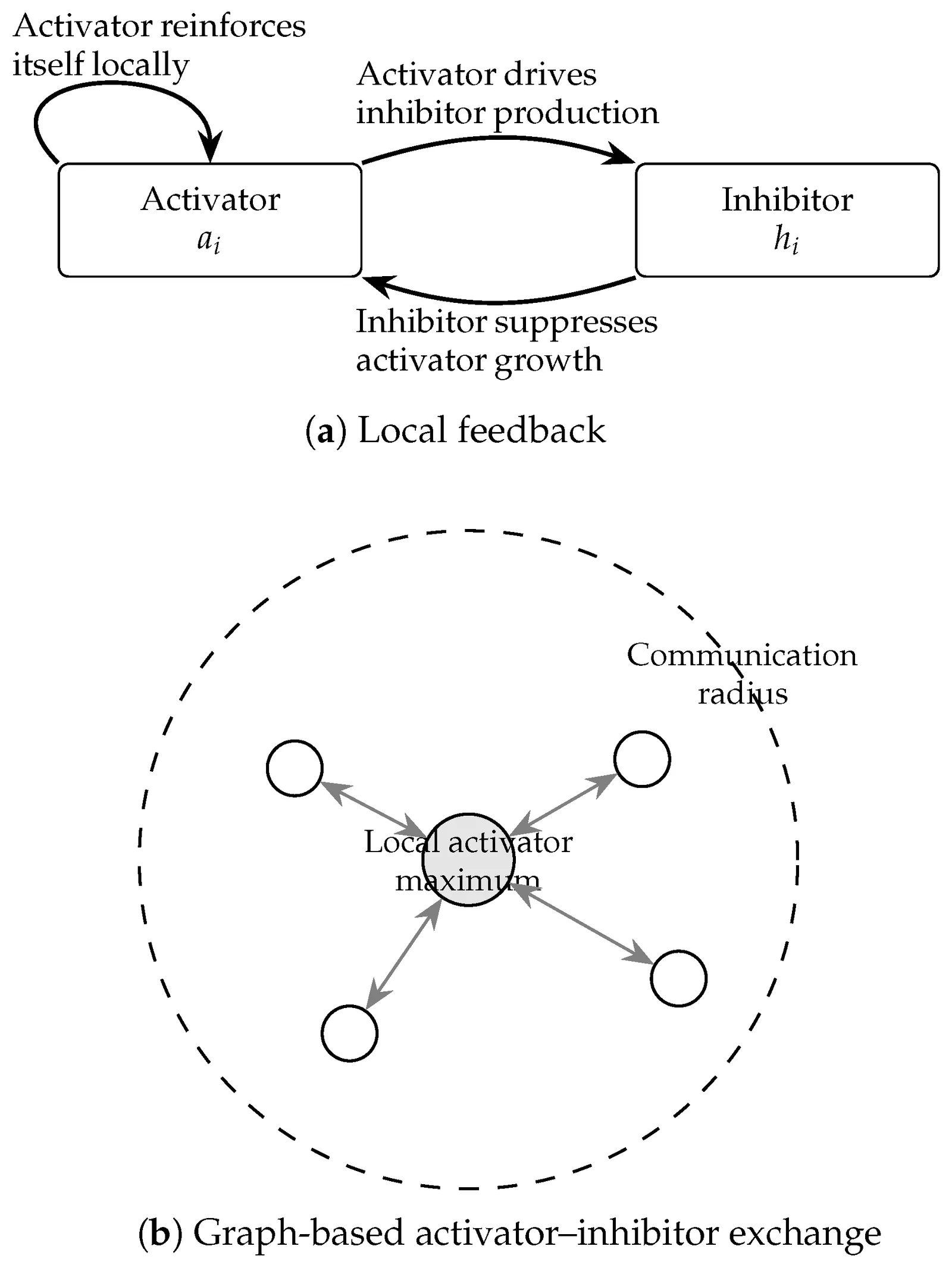
Basic activator–inhibitor mechanism used by EARDC. (**a**) Local feedback between activator and inhibitor values. (**b**) Graph-based activator–inhibitor exchange among one-hop neighbors within communication range. CH selection compares activator values within the CH separation radius $R_{\mathrm{CH}}$.

EARDC adopts the Gierer–Meinhardt activator–inhibitor model [53,54] as the basis for CH selection. Each sensor stores node-specific activator and inhibitor values $a_{i}$ and $h_{i}$. The continuous equations are presented first, followed by the graph-based discrete implementation used in EARDC:

(3) $$\frac{\partial a}{\partial t}=\frac{\sigma a^{2}}{h}-\mu_{a}a+\rho_{a}+D_{a}\nabla^{2}a,$$

(4) $$\frac{\partial h}{\partial t}=\sigma a^{2}-\mu_{h}h+\rho_{h}+D_{h}\nabla^{2}h.$$

where $\rho_{a}$ and $\rho_{h}$ are production rates, $\mu_{a}$ and $\mu_{h}$ are decay rates, $D_{a}$ and $D_{h}$ are diffusion coefficients, and $\nabla^{2}$ is the Laplacian operator. The parameter σ controls activator self-reinforcement and inhibitor production. The condition $D_{h}\gg D_{a}$ causes the inhibitor to spread more rapidly than the activator, which helps limit nearby activator maxima. Figure 3 illustrates this mechanism.

EARDC implements activator–inhibitor exchange over the sensor communication graph G(t)=(V(t),E(t)). The vertex set V(t) contains the alive sensors, and an edge in E(t) connects two alive sensors that can communicate directly at round *t*. Each sensor *i* stores activator and inhibitor values $a_{i}$ and $h_{i}$ and updates them using values received from alive one-hop neighbors.

The graph-based diffusion term is computed from the difference between a node’s value and the average value received from its one-hop neighbors. This operation replaces the continuous Laplacian in the WSN implementation. The updated activator value is used for the local comparison in CH selection, and the inhibitor and diffusion terms influence this update.

EARDC integrates residual energy into the activator–inhibitor update. Let $E_{i}\left(t\right)$ denote the residual energy of node *i* at round *t*. The normalized residual energy is defined as

(5) $${\hat{E}}_{i}\left(t\right)=\frac{E_{i}\left(t\right)}{E_{i}\left(0\right)}.$$

This normalization provides a dimensionless energy factor. The coefficient α controls the scaling strength, and the reaction and graph-based diffusion terms are multiplied by $\alpha {\hat{E}}_{i}\left(t\right)$. For the same local activator–inhibitor values and neighbor values, a larger ${\hat{E}}_{i}\left(t\right)$ gives a larger update magnitude. The energy-aware equations for node *i* are

(6) $$\frac{\partial a_{i}\left(t\right)}{\partial t}=\alpha {\hat{E}}_{i}\left(t\right)\left(\frac{\sigma a_{i}{\left(t\right)}^{2}}{h_{i}\left(t\right)}-\mu_{a}a_{i}\left(t\right)+\rho_{a}+D_{a}\nabla_{G}^{2}a_{i}\left(t\right)\right),$$

(7) $$\frac{\partial h_{i}\left(t\right)}{\partial t}=\alpha {\hat{E}}_{i}\left(t\right)\left(\sigma a_{i}{\left(t\right)}^{2}-\mu_{h}h_{i}\left(t\right)+\rho_{h}+D_{h}\nabla_{G}^{2}h_{i}\left(t\right)\right).$$

The parameters $\mu_{a}$, $\mu_{h}$, $\rho_{a}$, $\rho_{h}$, $D_{a}$, and $D_{h}$ have the same roles as in the original Gierer–Meinhardt activator–inhibitor model [53,54]. The continuous Laplacian $\nabla^{2}$ in Equations (3) and (4) is replaced in the implemented model by the graph diffusion operator $\nabla_{G}^{2}$, which is evaluated from one-hop value exchanges within the communication radius. This notation distinguishes the continuous reaction–diffusion model from its graph-based WSN implementation.

In the discrete implementation used in this work, the reaction–diffusion values are refreshed periodically rather than continuously. At each clustering epoch, all alive nodes synchronously update $a_{i}$ and $h_{i}$ using the activator and inhibitor values available before the current update. This update occurs every $T_{\mathrm{epoch}}$ rounds. The discrete update equations are

(8) $$a_{i}\left(t+T_{\mathrm{epoch}}\right)=a_{i}\left(t\right)+T_{\mathrm{epoch}}\;\alpha {\hat{E}}_{i}\left(t\right)\left(\frac{\sigma a_{i}{\left(t\right)}^{2}}{h_{i}\left(t\right)}-\mu_{a}a_{i}\left(t\right)+\rho_{a}+D_{a}\nabla_{G}^{2}a_{i}\left(t\right)\right),$$

(9) $$h_{i}\left(t+T_{\mathrm{epoch}}\right)=h_{i}\left(t\right)+T_{\mathrm{epoch}}\;\alpha {\hat{E}}_{i}\left(t\right)\left(\sigma a_{i}{\left(t\right)}^{2}-\mu_{h}h_{i}\left(t\right)+\rho_{h}+D_{h}\nabla_{G}^{2}h_{i}\left(t\right)\right).$$

Diffusion is evaluated in discrete form on the communication graph as

(10) $$\nabla_{G}^{2}a_{i}\left(t\right)=\frac{1}{|N_{i}|}\sum_{j\in N_{i}}a_{j}\left(t\right)-a_{i}\left(t\right),\;\nabla_{G}^{2}h_{i}\left(t\right)=\frac{1}{|N_{i}|}\sum_{j\in N_{i}}h_{j}\left(t\right)-h_{i}\left(t\right),$$

where $N_{i}$ is the set of alive one-hop neighbors of node *i* within $R_{\mathrm{comm}}$, and $|N_{i}|$ is the corresponding neighbor count. If $N_{i}$ is empty, the diffusion terms are set to zero, and the node is updated using only its local reaction terms. Because all nodes use values available before the current update, the updates are synchronous and independent of node-processing order.

Table 2 summarizes the per-sensor state maintained by EARDC. These variables support activator–inhibitor updates, CH selection, cluster membership, and sleep-state control. They do not require global topology information, exact node coordinates, or a centralized coverage map.

EARDC performs CH selection in a decentralized, self-organizing, and coordinate-free manner. Each node uses its activator value $a_{i}$, residual energy, sleep state, local range estimates, and activator values received from one-hop neighbors. The range estimates can be derived from RSSI or similar link-level measurements without exact node coordinates. CH selection therefore does not require a global topology map, geometric partitioning, or decisions computed at the base station.

At each clustering epoch, every alive node broadcasts its activator value to alive one-hop neighbors and compares the received values within the CH separation radius $R_{\mathrm{CH}}$. The communication radius $R_{\mathrm{comm}}$ defines the one-hop exchange range. When $R_{\mathrm{CH}}=R_{\mathrm{comm}}$, the same range is used for message exchange and CH selection. Smaller values related to the sensing radius can be used to obtain a denser CH set. An alive node with a locally maximal activator value within $R_{\mathrm{CH}}$ is selected as a CH. The selected CH announces its role and receives join requests during the same clustering epoch.

**Figure 4:**
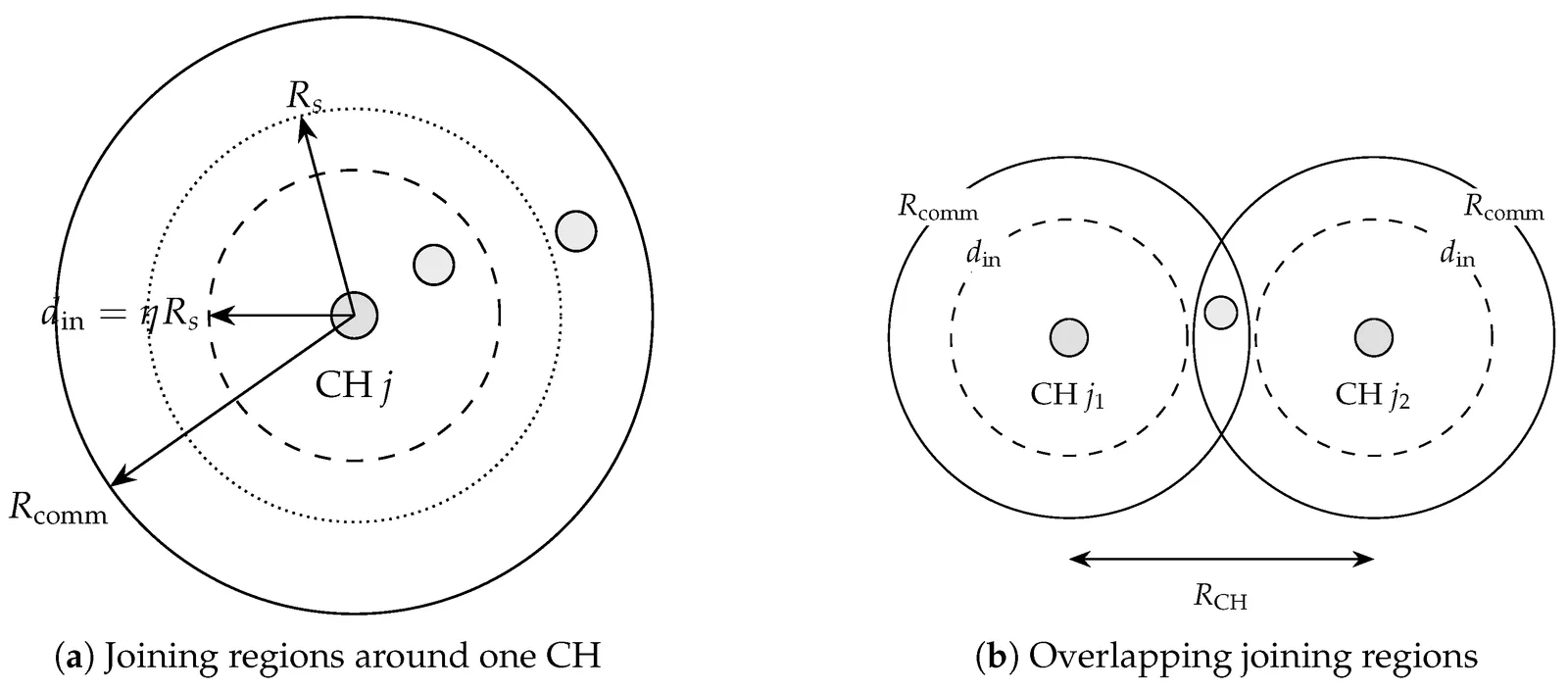
Radius relationships used for CH selection and cluster joining. (**a**) The inner joining distance $d_{\mathrm{in}}$, sensing radius $R_{s}$, and communication radius $R_{\mathrm{comm}}$ define the inner and outer regions around a CH. (**b**) A non-CH node may lie within the joining regions of two CHs. The radius $R_{\mathrm{CH}}$ specifies the CH separation distance.

After CH selection, non-CH nodes make local joining decisions based on node–CH distance, expected sensing overlap, local node degree, and residual energy. Let $R_{s}$ denote the sensing radius, and let $d_{ij}$ be the locally estimated distance between non-CH node *i* and CH *j*. The inner joining distance is defined as

(11) $$d_{\mathrm{in}}=\eta R_{s},$$

where 0<η<1 is a joining range factor. For CH *j*, node *i* lies in the inner region when $d_{ij}<d_{\mathrm{in}}$ and in the outer region when $d_{\mathrm{in}}\leq d_{ij}\leq R_{\mathrm{comm}}$. The inner region represents a high-overlap zone around the CH, where additional members are more likely to provide redundant sensing coverage. A node in the outer region has lower overlap with the CH sensing disk and can add coverage farther from the CH. Figure 4 shows these radius definitions together with the CH separation distance.

The joining range factor is set to η=0.8 from the geometric overlap between equal sensing disks. For two sensing disks with equal radius $R_{s}$ and center distance *d*, the pairwise overlap area is

(12) $$A_{\mathrm{overlap}}\left(d\right)=2R_{s}^{2}\cos^{-1}\;\left(\frac{d}{2R_{s}}\right)-\frac{d}{2}\sqrt{4R_{s}^{2}-d^{2}},$$

and the normalized overlap ratio is $\rho_{\mathrm{overlap}}\left(d\right)=A_{\mathrm{overlap}}\left(d\right)/\left(\pi R_{s}^{2}\right)$. At $d=0.8R_{s}$, $\rho_{\mathrm{overlap}}\left(d\right)\approx 0.50$. Thus, $d_{\mathrm{in}}=0.8R_{s}$ separates the inner region, where a node overlaps at least about half of the CH sensing disk, from the outer region, where a node can contribute coverage around the cluster boundary.

The inner region $d_{ij}<d_{\mathrm{in}}$ is not an unconditional joining region. Since a CH also performs sensing, a member located extremely close to the CH would create nearly duplicate sensing coverage and consume energy without adding meaningful area coverage. Inner region nodes therefore apply a probabilistic joining rule, and a node with $d_{ij}\leq 0.2R_{s}$ is excluded from requesting membership when other alive neighbors are available. This rule reduces redundant sensing near the CH.

The joining logic uses branch conditions before a join request is transmitted. If node *i* lies in the inner region of one CH and in the outer region of another CH, it remains unclustered for the current clustering epoch. This condition prevents ambiguous membership in overlapping joining regions.

If node *i* lies in the inner region of exactly one CH and does not lie in the outer region of any CH, that CH is selected for the membership decision. If $d_{ij}\leq 0.2R_{s}$ and node *i* has more than one alive neighbor, node *i* remains unclustered for the current clustering epoch. At this distance, the normalized disk overlap is approximately 0.87, which makes the node’s sensing coverage almost fully redundant with the CH sensing disk.

If node *i* does not lie in the inner region of any CH but lies in the outer region of one or more CHs, the node selects a CH according to local activator values. The shorter distance is used as a secondary criterion when needed. If no suitable CH remains, the node stays unclustered.

Both joining branches use a local crowding factor. Let

(13) $$d_{\mathrm{range}}=R_{\mathrm{comm}}-d_{\mathrm{in}},$$

and let $k_{i}$ be the number of alive neighbors of node *i* within distance $d_{\mathrm{range}}$. The expected number of neighbors within radius $d_{\mathrm{range}}$ under deployment density λ is

(14) $$k_{\exp}=\lambda \pi d_{\mathrm{range}}^{2}.$$

The local crowding factor is

(15) $$p_{0,i}=\min\left(1,\frac{k_{\exp}}{k_{i}+k_{\exp}}\right).$$

This factor decreases as the local node degree $k_{i}$ increases and reduces join attempts in areas with more neighboring nodes.

In the inner region branch, let *j* be the selected CH for node *i*, with $r_{i}=d_{ij}/d_{\mathrm{in}}$ and $q_{i}=d_{ij}/R_{s}$. The join probability is

(16) $$p_{\mathrm{inner},i}=\left\{\begin{array}{cc} \min\;\left(1,\frac{p_{0,i}}{2}\left(q_{i}r_{i}+\left(1-q_{i}\right){\hat{E}}_{i}\right)\right),\; & q_{i}\geq 0.5,\\ \min\;\left(1,\frac{p_{0,i}}{2}\left(\left(1-q_{i}\right)r_{i}+q_{i}{\hat{E}}_{i}\right)\right),\; & q_{i}<0.5. \end{array}\right.$$

Within the inner region, nodes farther from the CH and with smaller local node degree have a higher probability of sending a join request. Nodes closer to the CH have lower joining probability because their sensing disks overlap more strongly with the CH sensing disk. Residual energy can increase this probability when the too-close exclusion rule is not triggered. In the outer region branch, let *j* be the selected CH. With 

(17) $$s_{i}=\frac{d_{ij}-d_{\mathrm{in}}}{R_{\mathrm{comm}}-d_{\mathrm{in}}},$$

the join probability is

(18) $$p_{\mathrm{outer},i}=\min\;\left(1,p_{0,i}\left(\frac{d_{ij}}{R_{\mathrm{comm}}}+s_{i}{\hat{E}}_{i}\right)\right).$$

Under comparable crowding and residual energy conditions, the outer region rule generally assigns a higher join-request probability than the inner region rule. This reflects the lower expected overlap of outer region nodes with the CH sensing disk and their potential contribution near the cluster boundary. The computed probability determines whether node *i* transmits a join request to the selected CH. Each CH then evaluates received requests using local membership-control conditions. When alive nodes are available in the outer region $\left(d_{\mathrm{in}},R_{\mathrm{comm}}\right]$, the CH preferentially accepts outer region requesters because they add coverage with lower overlap near the cluster boundary. Inner region nodes are less preferred because they tend to duplicate the CH sensing disk. Requesting nodes that are not accepted receive rejection notifications, and accepted nodes record the selected CH for the current clustering epoch. Rejected and otherwise unclustered non-CH nodes enter a temporary sleep state until the next scheduled clustering epoch.

The inner- and outer-region conflict rule, too-close exclusion, and sleep-state rule form one local membership-control mechanism. CH selection establishes the CH set, and the membership and sleep rules determine which non-CH nodes remain active. This mechanism limits unnecessary sensing overlap after CH selection and reduces the associated energy consumption.

The CH selection and cluster formation procedures are decentralized. Algorithm 1 summarizes local-maximum CH selection, region-based cluster joining, and sleep state control.
**Algorithm 1** Decentralized EARDC cluster formation.  1:**Input:** alive nodes, sleep states, activator values $a_{i}$, residual energies ${\hat{E}}_{i}$, radii $R_{\mathrm{CH}},R_{s},R_{\mathrm{comm}}$  2:Set $d_{\mathrm{in}}\leftarrow \eta R_{s}$  3:**CH selection:**  4:**for all** alive nodes *i* **do**  5:      Obtain activator values from alive neighbors within $R_{\mathrm{CH}}$  6:      **if** $a_{i}$ is the local maximum within $R_{\mathrm{CH}}$ **then**  7:           Declare node *i* as a cluster head  8:           Advertise CH status to alive neighbors  9:      **end if**10:**end for**11:**Cluster joining:**12:**for all** alive non-CH nodes *i* **do**13:      Identify CHs for which node *i* lies in the inner region $\left(d_{ij}<d_{\mathrm{in}}\right)$ or outer region $\left(d_{\mathrm{in}}\leq d_{ij}\leq R_{\mathrm{comm}}\right)$14:      Compute $d_{\mathrm{range}}\leftarrow R_{\mathrm{comm}}-d_{\mathrm{in}}$, $k_{i}$, $k_{\exp}$, and $p_{0,i}$15:      **if** both inner region and outer region conditions are observed **then**16:             Keep node *i* unclustered for this clustering epoch17:      **else if** node *i* lies in the inner region of exactly one CH **then**18:             **if** $d_{ij}\leq 0.2R_{s}$ and *i* has more than one alive neighbor **then**19:                   Keep node *i* unclustered to avoid redundant overlap with CH sensing20:             **else**21:                   Compute $p_{\mathrm{inner},i}$ from $d_{ij}/d_{\mathrm{in}}$, $d_{ij}/R_{s}$, $p_{0,i}$, and ${\hat{E}}_{i}$22:                   Send a join request to the CH with probability $p_{\mathrm{inner},i}$23:             **end if**24:      **else if** node *i* lies in the outer region of one or more CHs **then**25:             Select a CH *j* using the largest activator value, with distance as a secondary criterion26:             Compute $p_{\mathrm{outer},i}$ from $d_{ij}/R_{\mathrm{comm}}$, $\left(d_{ij}-d_{\mathrm{in}}\right)/\left(R_{\mathrm{comm}}-d_{\mathrm{in}}\right)$, $p_{0,i}$, and ${\hat{E}}_{i}$27:             Send a join request to CH *j* with probability $p_{\mathrm{outer},i}$28:      **else**29:             Keep node *i* unclustered30:      **end if**31:**end for**32:**for all** cluster heads *c* **do**33:      Process received join requests using local membership-control conditions34:      **if** join requests are received from outer region nodes **then**35:             Prefer outer region requesters and reject redundant inner region requesters36:      **end if**37:      Send rejection notifications to requesting nodes that are not accepted38:**end for**39:For each unclustered non-CH node *i*, set $\tau_{i}\left(t\right)\leftarrow T_{\mathrm{epoch}}$ and place the node in sleep mode until the next scheduled clustering epoch

### 3.3. Localized Activation and Sleep-State Control

EARDC determines the active sensing set from selected CHs and accepted cluster memberships rather than from a separate global activation scheduler. The active sensing set is

(19) $$A\left(t\right)=\{i:i\;\mathrm{is}\;\mathrm{alive},\;\tau_{i}\left(t\right)=0,\;\mathrm{and}\;\left(i\in H\left(t\right)\;\mathrm{or}\;c_{i}\left(t\right)\neq ⌀\right)\},$$

where $\tau_{i}\left(t\right)$ is the remaining sleep timer, $\tau_{i}\left(t\right)=0$ means that node *i* is not sleeping, H(t) is the CH set, and $c_{i}\left(t\right)$ is the accepted cluster assignment of node *i*. Nodes outside A(t) do not contribute to coverage or data transmission energy consumption in that round. Sleeping nodes are also excluded from control-message exchange until the next scheduled clustering epoch.

A non-CH node becomes active only after it is accepted by a CH; being within communication range is not sufficient. Requesters that are not accepted receive rejection notifications, and rejected or otherwise unclustered non-CH nodes enter a temporary sleep state until the next scheduled clustering epoch. Thus, A(t) contains only selected CHs and accepted members. Sleeping nodes are excluded from sensing and data transmission energy costs.

### 3.4. Integration of Reaction–Diffusion Clustering and Active Sensing Control

This subsection specifies the ordering of CH selection, cluster membership, active sensing, and energy updates.

At each round, EARDC first updates sleep timers and removes nodes whose residual energy is below the operational threshold. At a clustering epoch, all alive nodes synchronously update their activator and inhibitor values, and Algorithm 1 updates the CH set, accepted cluster assignments, and sleep states. Between clustering epochs, the CH and membership states remain unchanged except when CHs or members deplete their energy.

After the cluster state is updated, the active sensing set A(t) is evaluated according to Section 3.3. Coverage, overlap, redundancy, and active ratio are computed from A(t). Sensing, control, and data transmission costs then update residual energy for the next round. Algorithm 2 summarizes this ordering, and Algorithm 1 specifies the local rules used at clustering epochs.

### 3.5. Coverage and Performance Metrics

This subsection defines the metrics used to evaluate sensing quality, redundancy, energy depletion, and cluster stability. All coverage-related quantities are computed from the active sensing set A(t) defined in Section 3.3. Nodes outside A(t) do not contribute to coverage or redundancy at round *t*.

Coverage is evaluated under a binary disk sensing model. Let G denote the set of grid cells used to discretize the deployment area, and let $n_{g}\left(t\right)$ be the number of active sensors whose sensing disks contain cell g∈G. The instantaneous coverage ratio is

(20) $$R_{c}\left(t\right)=\frac{\left|\{g\in G:n_{g}\left(t\right)>0\}\right|}{\left|G\right|}.$$

For a simulation trace of *T* rounds, the average coverage ratio is

(21) $${\bar{R}}_{c}=\frac{1}{T}\sum_{t=1}^{T}R_{c}\left(t\right).$$

Redundancy is computed from the same grid counts. Let $G_{\mathrm{cov}}\left(t\right)=\left\{g\in G:n_{g}\left(t\right)>0\right\}$. The overlap ratio is the fraction of covered cells with at least two active sensors:

(22) $$R_{\mathrm{overlap}}\left(t\right)=\frac{\left|\{g\in G_{\mathrm{cov}}\left(t\right):n_{g}\left(t\right)>1\}\right|}{|G_{\mathrm{cov}}\left(t\right)|},$$

with $R_{\mathrm{overlap}}\left(t\right)=0$ when $G_{\mathrm{cov}}\left(t\right)$ is empty. The redundancy degree is the average number of additional active sensors covering a covered grid cell:

(23) $$D_{\mathrm{red}}\left(t\right)=\frac{1}{|G_{\mathrm{cov}}\left(t\right)|}\sum_{g\in G_{\mathrm{cov}}\left(t\right)}\left(n_{g}\left(t\right)-1\right).$$

The redundant node ratio is computed over active nodes:

(24) $$R_{\mathrm{node}}\left(t\right)=\frac{N_{\mathrm{red}}\left(t\right)}{\left|A(t)\right|},$$

where $N_{\mathrm{red}}\left(t\right)$ is the number of active nodes whose covered grid cells are all also covered by at least one other active node. If there are no covered cells or no active nodes, the corresponding redundancy metric is set to zero.
**Algorithm 2** Round-level EARDC execution.  1:**Initialize:** Deploy *N* sensors by a PPP over area *A*.  2:Set clustering-epoch period $T_{\mathrm{epoch}}$  3:**for all** nodes *i* **do**  4:      $E_{i}\leftarrow E_{0}$; initialize activator $a_{i}$, inhibitor $h_{i}$, and cluster state; set $\tau_{i}\left(0\right)\leftarrow 0$  5:**end for**  6:Initialize round t←0, CH set H(t)←⌀, cluster set C(t)←⌀, and active set A(t)←⌀  7:**while** at least one node is alive **do**  8:      Increment round: t←t+1  9:      Decrement each positive sleep timer by one; identify alive nodes above the operational threshold10:      **if** $t\;\mathrm{mod}\;T_{\mathrm{epoch}}=0$ **then**11:             Use activator and inhibitor values from the previous update interval12:             **for all** alive nodes *i* **do**13:                   Update $a_{i},h_{i}$ and compute ${\hat{E}}_{i}$14:             **end for**15:             Remove dead cluster heads from H(t) and dead members from C(t)16:             Invoke Algorithm 1 to update H(t), C(t), and sleep states17:      **else**18:             Use the current CH and membership states after removing dead CHs or members19:      **end if**20:      **Assign activity state for coverage and data transmission:**21:      Reset A(t)←⌀22:      **for all** alive nodes *i* **do**23:             **if** *i* is sleeping **then**24:                   Mark *i* inactive25:             **else if** i∈H(t) or *i* belongs to some cluster in C(t) **then**26:                   Mark *i* active27:                   Add *i* to A(t)28:             **else**29:                   Mark *i* inactive30:             **end if**31:      **end for**32:      Compute coverage and redundancy metrics using A(t) only33:      **Charge data transmission energy:**34:      Nodes in A(t)∩{i:i∉H(t)} sense and transmit one packet to their CHs35:      CHs in A(t)∩H(t) sense, receive member packets, aggregate data, and transmit to the base station36:      Orphan CHs in A(t)∩H(t) sense and transmit directly to the base station37:      Clamp depleted node energy to zero and remove nodes below the operational threshold38:      Record alive ratio, |A(t)|/N, |H(t)|, coverage, overlap, and redundancy at round *t*39:**end while**

Energy and stability metrics are recorded over the same round sequence. Network lifetime is the last round before all nodes fall below the operational energy threshold:

(25) $$T_{\mathrm{life}}=\max\left\{t:N_{\mathrm{alive}}\left(t\right)>0\right\}.$$

First node death (FND) is

(26) $$T_{\mathrm{FND}}=\min\left\{t:N_{\mathrm{alive}}\left(t\right)<N_{\mathrm{initial}}\right\}.$$

For a coverage threshold θ∈(0,1], the coverage-retention time is the first round in which the coverage ratio falls below that threshold:

(27) $$T_{\mathrm{cov}}\left(\theta \right)=\min\left\{t:R_{c}\left(t\right)<\theta \right\}.$$

If coverage is initially below θ, $T_{\mathrm{cov}}\left(\theta \right)=0$; if it remains above the threshold, the network lifetime is used. The coverage-bounded lifetime counts the rounds in which the coverage requirement is satisfied:

(28) $$L_{\mathrm{cov}}\left(\theta \right)=\sum_{t=0}^{T_{\mathrm{life}}}1\;\left\{R_{c}\left(t\right)\geq \theta \right\},$$

where 1{·} is the indicator function. Cluster stability is measured as the temporal standard deviation of the number of clusters:

(29) $$S_{\mathrm{cluster}}=\mathrm{std}\;\left(|H(t)|\right).$$

Table 3 summarizes the coverage and performance metrics used in the analysis and simulations.

## 4. Theoretical Analysis of the Energy-Aware Reaction–Diffusion Dynamics

This section analyzes the energy-aware reaction–diffusion component of EARDC before the local CH-selection rule is applied. The homogeneous-energy analysis examines the equilibrium, linear stability, growth rate, and characteristic wavelength of the activator–inhibitor update. The characteristic wavelength provides a reference for the reaction–diffusion mechanism and is not used to prescribe an energy-dependent CH spacing.

The theoretical characterization is limited to the activator–inhibitor update. In the implemented protocol, alive nodes with local activator maxima within $R_{\mathrm{CH}}$ are selected as CHs according to Algorithm 1; cluster membership and sleep-state control are applied afterward.

The analysis follows the variables introduced in Section 3. At node *i*, normalized residual energy ${\hat{E}}_{i}\left(t\right)$, scaled by α, multiplies the local reaction and graph-based diffusion terms. Under uniform residual energy, this multiplier changes the rate at which perturbations grow or decay. It leaves the homogeneous equilibrium and the classical diffusion-driven instability condition unchanged. Under nonuniform residual energy, the multiplier varies among nodes and changes their relative update rates. The homogeneous analysis does not establish a specific relationship between this variation and CH spacing.

### 4.1. Homogeneous Steady-State and Linearization

Consider a spatially homogeneous state with constant normalized energy ${\hat{E}}_{i}\left(t\right)\equiv {\hat{E}}_{0}>0$. This setting represents either an early deployment stage or a local region with nearly uniform residual energy. Let $a_{i}\left(t\right)=a^{*}$ and $h_{i}\left(t\right)=h^{*}$ for all nodes. Since the Laplacian terms vanish in this state, the equilibrium is determined by the reaction terms:

(30) $$\begin{array}{cc} \alpha {\hat{E}}_{0}\left(\frac{\sigma {\left(a^{*}\right)}^{2}}{h^{*}}-\mu_{a}a^{*}+\rho_{a}\right) & =0, \end{array}$$

(31) $$\begin{array}{cc} \alpha {\hat{E}}_{0}\left(\sigma {\left(a^{*}\right)}^{2}-\mu_{h}h^{*}+\rho_{h}\right) & =0. \end{array}$$

Since $\alpha {\hat{E}}_{0}>0$, the steady state satisfies

(32) $$\begin{array}{cc} \frac{\sigma {\left(a^{*}\right)}^{2}}{h^{*}}-\mu_{a}a^{*}+\rho_{a} & =0, \end{array}$$

(33) $$\begin{array}{cc} \sigma {\left(a^{*}\right)}^{2}-\mu_{h}h^{*}+\rho_{h} & =0. \end{array}$$

Solving Equation (33) for $h^{*}$ gives

(34) $$h^{*}=\frac{\sigma {\left(a^{*}\right)}^{2}+\rho_{h}}{\mu_{h}}.$$

Substituting this expression into (32) yields a nonlinear equation in $a^{*}$. An explicit algebraic solution is not needed for the stability analysis; $a^{*}$ and $h^{*}$ can be obtained numerically for a given parameter set.

To linearize around the equilibrium, write $a_{i}\left(t\right)=a^{*}+u_{i}\left(t\right)$ and $h_{i}\left(t\right)=h^{*}+v_{i}\left(t\right)$, where $u_{i}$ and $v_{i}$ are small perturbations. After higher-order terms are omitted, the activator expansion is

(35) $$\begin{array}{cc} \frac{\sigma {\left(a^{*}+u\right)}^{2}}{h^{*}+v}-\mu_{a}\left(a^{*}+u\right)+\rho_{a} & \approx \left(\frac{2\sigma a^{*}}{h^{*}}-\mu_{a}\right)u-\frac{\sigma {\left(a^{*}\right)}^{2}}{{\left(h^{*}\right)}^{2}}v, \end{array}$$

where constant terms cancel due to the steady-state conditions. Similarly, for the inhibitor equation,

(36) $$\begin{array}{cc} \sigma {\left(a^{*}+u\right)}^{2}-\mu_{h}\left(h^{*}+v\right)+\rho_{h} & \approx 2\sigma a^{*}\;u-\mu_{h}v. \end{array}$$

The diffusion terms become $\nabla^{2}u$ and $\nabla^{2}v$ because $a^{*}$ and $h^{*}$ are spatially constant. The linearized system is therefore

(37) $$\frac{\partial }{\partial t}\left[\begin{array}{c} u\\ v \end{array}\right]=\alpha {\hat{E}}_{0}\left(J\left[\begin{array}{c} u\\ v \end{array}\right]+\left[\begin{array}{c} D_{a}\nabla^{2}u\\ D_{h}\nabla^{2}v \end{array}\right]\right),$$

where *J* is the Jacobian matrix of the reaction terms evaluated at the steady state $\left(a^{*},h^{*}\right)$:

(38) $$J=\left[\begin{array}{cc} f_{u} & f_{v}\\ g_{u} & g_{v} \end{array}\right],$$

The entries are

(39) $$\begin{array}{cccc} f_{u} & =\frac{2\sigma a^{*}}{h^{*}}-\mu_{a}, & f_{v} & =-\frac{\sigma {\left(a^{*}\right)}^{2}}{{\left(h^{*}\right)}^{2}}, \end{array}$$

(40) $$\begin{array}{cccc} g_{u} & =2\sigma a^{*}, & g_{v} & =-\mu_{h}. \end{array}$$

The Jacobian *J* describes the local reaction response around the homogeneous operating point. If its eigenvalues have negative real parts, a perturbation at a node decays when diffusion is absent. The uniform energy factor $\alpha {\hat{E}}_{0}$ only multiplies this decay or growth rate. Homogeneous energy changes the speed of convergence, not the sign of the reaction stability condition. Adding spatial modes determines whether local stability can be destabilized by diffusion and form separated activator maxima.

### 4.2. Dispersion Relation and Turing Instability

This subsection examines whether diffusion destabilizes the reaction-stable homogeneous state. Let ω denote the temporal growth rate of a perturbation with spatial wavenumber *k*, distinct from the deployment density λ. In the continuous aggregate setting, perturbations have the form $e^{\omega t+ikx}$. In the implemented graph setting, the analogous modes are communication-graph Laplacian eigenvectors; $k^{2}$ is interpreted as the corresponding eigenvalue scale. Under the continuous notation, the growth rate satisfies

(41) $$\omega \left(k\right)=\alpha {\hat{E}}_{0}\;\frac{1}{2}\left[\mathrm{Tr}\left(J_{k}\right)\pm \sqrt{\mathrm{Tr}{\left(J_{k}\right)}^{2}-4\det\left(J_{k}\right)}\right],$$

where $\alpha {\hat{E}}_{0}$ is the uniform energy-coupling factor under homogeneous conditions, $J_{k}$ is the diffusion-modified Jacobian evaluated at the steady state, and Tr(·) and det(·) denote the trace and determinant, respectively. The diffusion-modified Jacobian $J_{k}$ is given by

(42) $$J_{k}=\left[\begin{array}{cc} f_{u}-D_{a}k^{2} & f_{v}\\ g_{u} & g_{v}-D_{h}k^{2} \end{array}\right].$$

Here, $\left(f_{u},f_{v},g_{u},g_{v}\right)$ are evaluated at $\left(a^{*},h^{*}\right)$.

A diffusion-driven (Turing) instability occurs when the homogeneous steady state is stable in the absence of diffusion but unstable for a finite range of spatial frequencies. The first requirement is reaction stability without diffusion, expressed by Tr(J)<0 and det(J)>0. The second requirement is that diffusion creates an unstable spatial mode: there must exist a nonzero wavenumber $k_{c}>0$ such that $ℜ[\omega \left(k_{c}\right)]>0$. For EARDC, the activator maxima used in CH selection depend on both local activation and inhibitor feedback rather than on unconstrained activator growth.

The characteristic wavenumber $k_{c}$, defined as the value that maximizes ℜ[ω(k)], determines the dominant spatial scale, with characteristic wavelength $\lambda_{c}=2\pi /k_{c}$. Under homogeneous energy, $\alpha {\hat{E}}_{0}$ uniformly scales ω(k). It changes convergence or growth speed, not the Turing conditions or $k_{c}$. In EARDC, $\lambda_{c}$ is an aggregate spacing scale for activator maxima; the implemented CH spacing is additionally shaped by $R_{\mathrm{CH}}$, local node placement, and the communication graph.

Nonuniform residual energy breaks the uniform-coefficient assumption used above. The homogeneous linearization is therefore a reference case rather than a complete stability analysis for networks with unequal node energy. No energy-dependent wavelength or CH-spacing relationship is inferred from this linearization.

## 5. Analysis of Coverage

The preceding section characterized the reaction–diffusion update under homogeneous residual energy. In EARDC, the selected CH set is determined by local activator maxima and the CH separation radius $R_{\mathrm{CH}}$. The active-node density then depends on the probability of CH selection and the conditional probability that a non-CH node remains active after membership acceptance and temporary sleep-state control. These probabilities provide aggregate descriptions of CH and member participation. The number of active sensors covering a location is approximated by a Poisson distribution whose mean is determined by the average active-node density. The resulting probabilities are used to characterize coverage, overlap, and redundancy.

The conditional non-CH participation probability summarizes the inner/outer region tests, probabilistic join requests, CH acceptance decisions, and temporary sleep states. Numerical evaluation of the active-node density expression requires this probability and the EARDC CH probability.

Geometric coverage analysis often uses sensor coordinates, pairwise distances, or the complete active-node layout. These assumptions support deterministic placement, geometric coverage optimization, and centralized scheduling. Individual nodes in EARDC do not require this network-wide information. The analysis therefore uses aggregate descriptions. A regular hexagonal reference layout gives geometric interpretation to the characteristic wavelength under homogeneous residual energy. For random deployment, the average active-node density is estimated from the active probability. Coverage is then estimated by approximating the number of active sensors covering each location with a Poisson distribution.

### 5.1. Analysis Under Hexagonal Packing

The hexagonal model is an idealized reference, not an EARDC deployment. It shows the geometric implication of a characteristic CH spacing before random node locations, graph constraints, and local sensing control are considered. In this reference case, $\lambda_{c}$ is interpreted as the center-to-center spacing between neighboring CHs. This spacing allows coverage load and overlap to be expressed in closed form.

Let the monitoring region be

(43) $$|A_{\mathrm{total}}|=L_{x}L_{y}$$

where $L_{x}$ and $L_{y}$ denote the length and width of the region, respectively.

**Figure 5:**
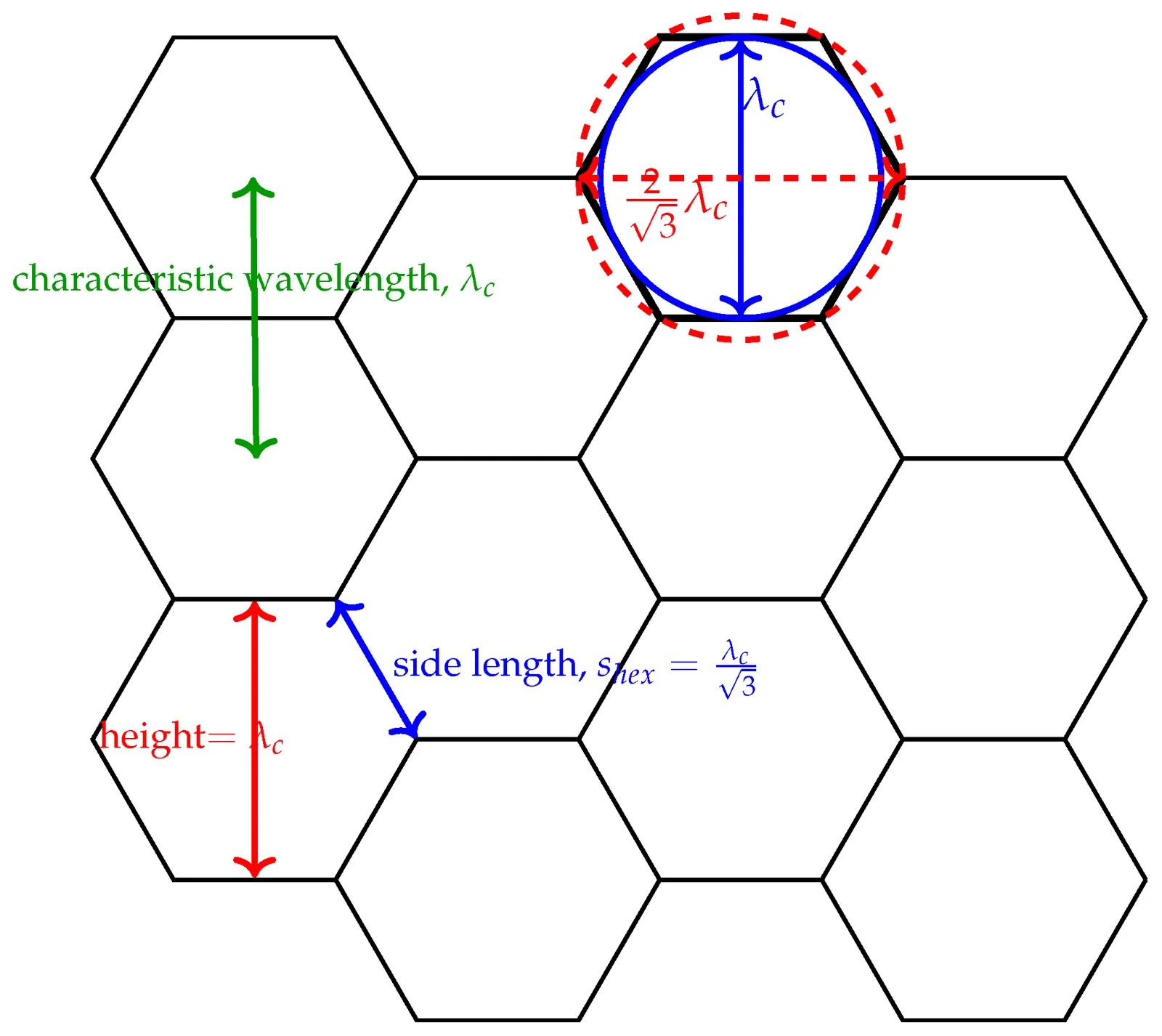
Regular hexagonal reference packing used to interpret the characteristic spacing $\lambda_{c}$. The highlighted cell shows the incircle (blue) and circumcircle (red, dashed).

In the reference layout, CHs form a triangular lattice and each CH is assigned a regular hexagonal cell, as shown in Figure 5. The area assigned to one CH is

(44) $$|A_{\mathrm{hex}}|=\frac{\sqrt{3}}{2}\lambda_{c}^{2}$$

where $\lambda_{c}$ is the center-to-center distance between neighboring CHs and represents the characteristic spacing associated with the reaction–diffusion update.

The total number of clusters in the domain is then approximately

(45) $$N_{s}\approx \frac{|A_{\mathrm{total}}|}{|A_{\mathrm{hex}}|}=\frac{2L_{x}L_{y}}{\sqrt{3}\lambda_{c}^{2}}$$

Each hexagonal cell is assigned an idealized circular sensing footprint of diameter $d_{s}$. The diameter ranges from the hexagon incircle diameter $\lambda_{c}$ to the circumcircle diameter $\frac{2}{\sqrt{3}}\lambda_{c}$, as shown in Figure 5. The footprint area is

(46) $$|A_{s}|=\frac{\pi d_{s}^{2}}{4}$$

The corresponding aggregate sensing-area load is

(47) $$\Gamma \left(d_{s}\right)=\frac{N_{s}\left|A_{s}\right|}{|A_{\mathrm{total}}|}\approx \frac{\pi d_{s}^{2}}{2\sqrt{3}\lambda_{c}^{2}}.$$

Table 4 summarizes the parameters used in the hexagonal reference analysis.

#### 5.1.1. Coverage Ratio

A feasible coverage ratio cannot exceed one. In the hexagonal reference model, the useful analytical quantity is therefore the aggregate sensing-area load $\Gamma (d_{s})$. Values below one indicate potential uncovered area under non-overlapping placement. Values above one indicate that sensing disks must overlap.

Under the circumcircle case $d_{s}\approx \frac{2}{\sqrt{3}}\lambda_{c}$, substitution into $\Gamma (d_{s})$ gives

$$\begin{array}{cc} \Gamma_{\max}\; & =\frac{\pi }{2\sqrt{3}\lambda_{c}^{2}}{\left(\frac{2}{\sqrt{3}}\lambda_{c}\right)}^{2}\\ \; & =\frac{2\sqrt{3}\pi }{9}\approx 1.209. \end{array}$$

The feasible coverage ratio is bounded by

(48) $$R_{c}\leq \min\left\{1,\Gamma_{\max}\right\}=1.$$

Thus, $\Gamma_{\max}>1$ is not a coverage percentage; it indicates excess sensing-area load in the idealized regular layout. EARDC uses membership and sleep-state control to limit unnecessary sensing participation associated with such overlap.

For the incircle case $d_{s}\approx \lambda_{c}$, the sensing-area load is

$$\begin{array}{cc} \Gamma_{\min} & =\frac{\pi }{2\sqrt{3}\lambda_{c}^{2}}\lambda_{c}^{2}=\frac{\pi }{2\sqrt{3}}\approx 0.907. \end{array}$$

This value indicates that non-overlapping incircle footprints leave part of each hexagonal cell uncovered. The interval $\Gamma_{\min}\leq \Gamma \left(d_{s}\right)\leq \Gamma_{\max}$ therefore summarizes the geometric tradeoff between possible gaps and redundant overlap.

#### 5.1.2. Overlap Ratio

In the circumcircle case, the aggregate sensing-area load exceeds one. If the union of the footprints covers the monitoring region, the excess is interpreted as overlap load counted with multiplicity in the regular reference layout.

The overlap-load fraction relative to aggregate sensed area is

(49) $$\rho_{\mathrm{hex}}=\frac{|A_{\mathrm{overlap}}|}{N_{s}\left|A_{s}\right|}.$$

Using $N_{s}|A_{s}|=\Gamma_{\max}\left|A_{\mathrm{total}}\right|$, the excess sensed area is

$$|A_{\mathrm{overlap}}|=\left(\Gamma_{\max}-1\right)\left|A_{\mathrm{total}}\right|.$$

Thus,

$$\begin{array}{cc} \rho_{\mathrm{hex}} & =\frac{\Gamma_{\max}-1}{\Gamma_{\max}}\\ \; & =1-\frac{9}{2\sqrt{3}\pi }\\ \; & \approx 0.173. \end{array}$$

This quantity is a reference load value for the ideal regular layout. It is not identical to the simulated overlap ratio, which is computed from active-node grid counts as defined in Section 3.5.

### 5.2. Analysis Under Random Deployment

At initial deployment, sensor locations follow a homogeneous PPP. EARDC subsequently selects CHs and active members through local CH competition, membership acceptance, and sleep-state control. These operations introduce dependence among the participation decisions of neighboring nodes. Consequently, the selected CHs and active nodes do not follow the initial PPP model.

#### 5.2.1. Active-Node Density and Effective Coverage

After CH selection, selected CHs and accepted members form the active sensing set. A non-CH node becomes active only if it sends a join request and the CH accepts that request. Other non-CH nodes sleep until the next scheduled clustering epoch. The analysis represents the combined effect of these protocol decisions through a CH-selection probability and a conditional non-CH participation probability.

Let $p_{\mathrm{CH}}\left(x,t\right)$ denote the average probability that a sensor near *x* is selected as a CH under the EARDC local-maximum rule. This probability depends on activator values, residual energy, local node placement, and $R_{\mathrm{CH}}$. Let $q_{\mathrm{MS}}\left(x,t\right)$ denote the conditional probability that an alive non-CH node belongs to the active sensing set after membership acceptance and temporary sleep-state control. This probability summarizes the inner/outer region tests, probabilistic join requests, CH acceptance decisions, and the resulting sleep states. The resulting active probability is approximated by

$$p_{\mathrm{act}}\left(x,t\right)\approx p_{\mathrm{CH}}\left(x,t\right)+\left(1-p_{\mathrm{CH}}\left(x,t\right)\right)q_{\mathrm{MS}}\left(x,t\right).$$

The average active-node density is estimated by

$$\lambda_{\mathrm{act}}\left(x,t\right)=\lambda p_{\mathrm{act}}\left(x,t\right).$$

This expression uses the average active probability and does not assume independent selection of active nodes from the initial PPP. CH selection and membership control introduce dependence among active-node locations. The active-node locations are therefore not assumed to form a Poisson process. For coverage calculation, the number of active sensors covering a location is approximated by a Poisson random variable whose mean is determined by the average active-node density $\lambda_{\mathrm{act}}\left(x,t\right)$. Under the binary disk model, let

$$m\left(x,t\right)=\int_{B\left(x,r_{s}\right)\cap A_{\mathrm{total}}}\lambda_{\mathrm{act}}\left(y,t\right)\;dy$$

denote the expected number of active sensors whose sensing disks cover location *x*, where $B(x,r_{s})$ is the disk of radius $r_{s}$ centered at *x*. The intersection with $A_{\mathrm{total}}$ accounts for sensing disks truncated by the deployment boundary. The point coverage probability is

$$P_{\mathrm{cov}}\left(x,t\right)=1-\exp\;\left(-m(x,t)\right).$$

The effective coverage ratio is therefore

$$R_{c,\mathrm{eff}}\left(t\right)=\frac{1}{|A_{\mathrm{total}}|}\int_{A_{\mathrm{total}}}\left(1-\exp\;\left(-m(x,t)\right)\right)dx.$$

For a homogeneous active-node density approximation, define the spatially averaged active-node density as

$${\bar{\lambda }}_{\mathrm{act}}\left(t\right)=\frac{1}{|A_{\mathrm{total}}|}\int_{A_{\mathrm{total}}}\lambda_{\mathrm{act}}\left(x,t\right)\;dx.$$

The expected number of active sensors covering location *x* is then

$$m\left(x,t\right)={\bar{\lambda }}_{\mathrm{act}}\left(t\right)\left|B\left(x,r_{s}\right)\cap A_{\mathrm{total}}\right|.$$

The intersection with $A_{\mathrm{total}}$ accounts for sensing disks truncated by the deployment boundary.

#### 5.2.2. Overlap Ratio and Redundancy

Under the same active-node density approximation, let $N_{x}$ denote the number of active sensors whose sensing disks cover location *x*. Approximating $N_{x}$ as Poisson with mean m(x,t), the overlap ratio is

$$\begin{array}{cc} \rho_{\mathrm{overlap}}\left(t\right) & =\frac{\int_{A_{\mathrm{total}}}P\left(N_{x}\geq 2\right)\;dx}{\int_{A_{\mathrm{total}}}P\left(N_{x}\geq 1\right)\;dx}\\ \; & =\frac{\int_{A_{\mathrm{total}}}\left(1-e^{-m(x,t)}-m\left(x,t\right)e^{-m(x,t)}\right)dx}{\int_{A_{\mathrm{total}}}\left(1-e^{-m(x,t)}\right)dx}. \end{array}$$

This expression is the continuous analogue of the grid overlap ratio in Section 3.5.

The pointwise redundant contribution is $d_{r}\left(x\right)={\left(N_{x}-1\right)}^{+}$, where ${\left(z\right)}^{+}=\max\left(0,z\right)$. For a Poisson random variable with mean m(x,t),

$$E\;\left[{\left(N_{x}-1\right)}^{+}\right]=m\left(x,t\right)-\left(1-e^{-m(x,t)}\right).$$

The redundancy degree over the covered region is therefore approximated by

$${\bar{d}}_{r}\left(t\right)\approx \frac{\int_{A_{\mathrm{total}}}\left[m\left(x,t\right)-\left(1-e^{-m(x,t)}\right)\right]dx}{\int_{A_{\mathrm{total}}}\left(1-e^{-m(x,t)}\right)dx},$$

when the denominator is nonzero. Node-level redundancy is evaluated numerically by checking whether every grid cell covered by an active node is also covered by another active node:

$$\rho_{r}\left(t\right)=\frac{N_{\mathrm{redundant}}\left(t\right)}{N_{\mathrm{active}}\left(t\right)}.$$

In this approximation, a lower $\lambda_{\mathrm{act}}\left(x,t\right)$ reduces expected overlap and redundancy. The analytical expressions describe aggregate active-node behavior rather than individual grid cells.

## 6. Simulation and Results

This section evaluates EARDC under random WSN deployment and compares it with clustering baselines. All simulations were conducted using a custom round-based simulator implemented in Python 3. Residual energy affects the activator–inhibitor update used for CH selection, and local membership and sleep-state decisions determine the active sensing set. The simulations examined network lifetime, active participation, coverage, overlap, redundancy, and cluster stability at three deployment densities.

### 6.1. Simulation Setup

Simulations were conducted over a square monitoring field of $100\times 100\;m^{2}$ with static sensor nodes deployed according to a homogeneous PPP. Three deployment densities were considered: λ=0.001, λ=0.005, and λ=0.02 nodes/$m^{2}$, corresponding to sparse, moderately dense, and dense deployments.

**Figure 6:**
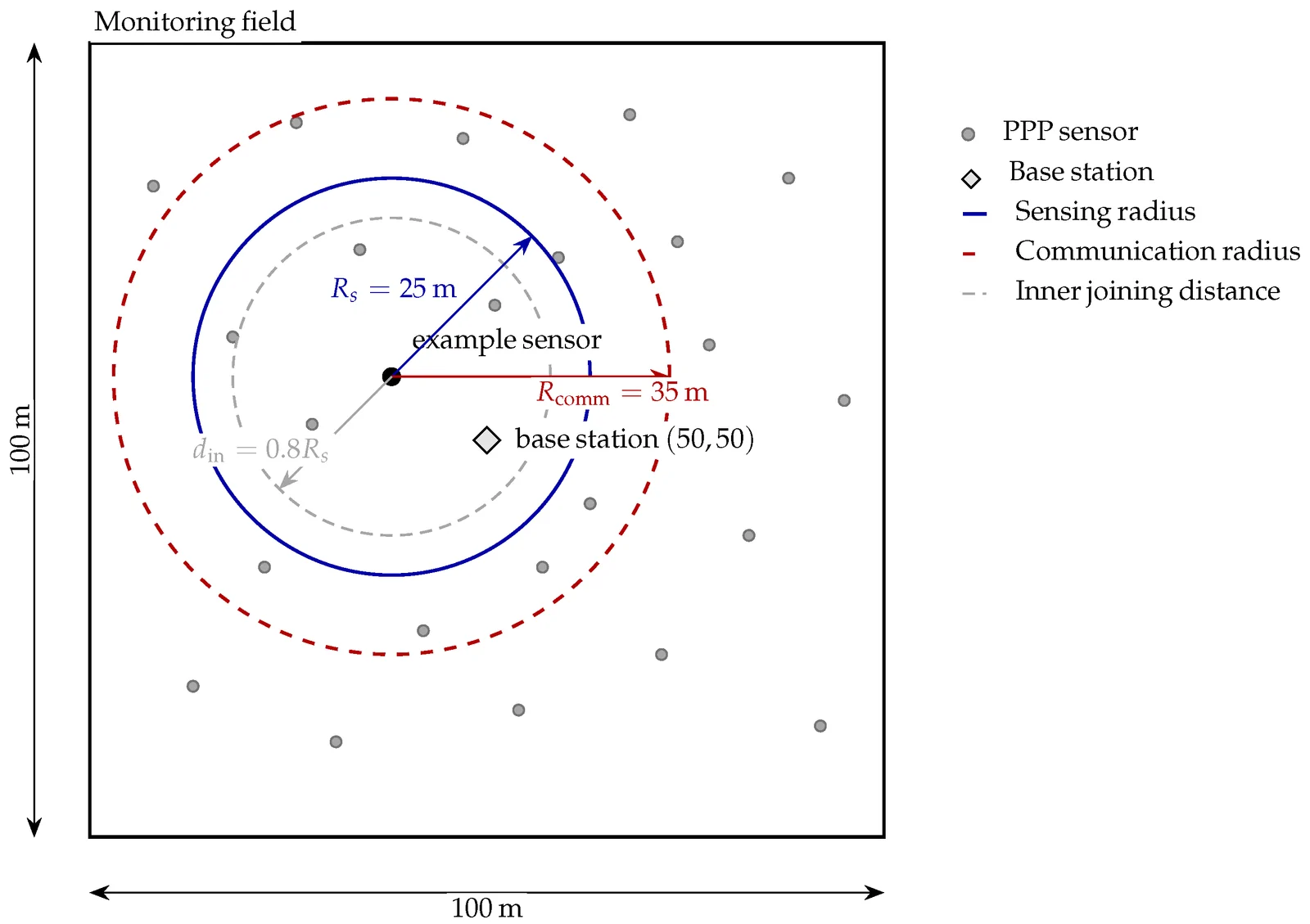
Simulation deployment geometry.

Each sensor node was initialized with 1J of energy. The base station was located at the center of the monitoring field, (50,50), and all nodes were static. The communication radius was 35m, and the sensing radius was 25m. Figure 6 summarizes the monitoring field, base-station placement, and the radius relationships used in the simulation.

Each simulation run used an independently generated homogeneous PPP deployment. Sensor positions remained fixed after deployment, and the base station remained at the field center. The three densities corresponded to expected node populations of 10, 50, and 200 in the $100\times 100\;m^{2}$ field. The fixed communication radius determined one-hop connectivity, activator–inhibitor exchange, and the candidate neighborhoods used during cluster formation. Base-station placement determined CH-to-base-station transmission distances and the corresponding radio-energy costs.

The measured active ratio was the simulation quantity associated with the active probability $p_{\mathrm{act}}\left(x,t\right)$. Coverage, overlap, and redundancy were evaluated on the coverage grid from the active sensing set over the network lifetime of each protocol. Coverage retention and coverage-bounded lifetime were evaluated at θ∈{0.9,0.8,0.7,0.6,0.5}.

Energy consumption was modeled using the first-order radio model commonly adopted in clustered WSN studies. The corresponding radio parameters, packet sizes, control message sizes, and reaction–diffusion message sizes are summarized in Table 5, along with the principal parameters used in the simulations.

The control overhead of EARDC was included in the same radio energy model through the message sizes listed in Table 5. This overhead came from periodic local exchanges during cluster formation, including the exchange of activator and inhibitor values among one-hop neighbors, CH announcements, join requests, and rejection messages. These messages were confined to the local communication range. The base station did not collect global network state, compute clustering decisions, or disseminate optimization decisions for EARDC.

At each clustering epoch, the simulator applied one synchronous activator–inhibitor update using α=0.01 and $T_{\mathrm{epoch}}=20$, for which $\alpha T_{\mathrm{epoch}}=0.2$. Negative numerical values were set to zero after the update, and $\epsilon_{h}=10^{-6}$ was used when the inhibitor denominator was zero. The activator–inhibitor equations were not iterated to a convergence tolerance, and no internal stopping criterion was used.

For the normalized graph diffusion operator in Equation (10), the eigenvalues lie in [−2,0]. Since $0\leq {\hat{E}}_{i}\left(t\right)\leq 1$, the linear decay and graph diffusion terms satisfied the conservative explicit-update condition $\alpha T_{\mathrm{epoch}}\left(\mu_{x}+2D_{x}\right)\leq 2$ for x∈{a,h}. The corresponding values were 0.022 for the activator and 0.12 for the inhibitor. This check concerned the linear terms and did not establish global convergence of the nonlinear activator–inhibitor update.

The simulation study compared clustering and active sensing behavior rather than routing optimization. After cluster formation and data aggregation, each CH transmitted its aggregated data directly to the base station; inter-CH relay and gateway-assisted multi-hop routing were not enabled. Active nodes forwarded sensing data to their CHs in each round rather than only after a detected event. This common data transmission model prevents routing differences from confounding the comparison of CH selection, cluster membership, coverage maintenance, and redundancy control. The centered base-station location was identical for all protocols and held sink placement constant in the comparison. Multi-hop routing can be coupled with EARDC in larger monitoring fields or off-center sink deployments. This extension is outside the scope of the present evaluation.

In EARDC, alive nodes with local activator maxima within the CH separation radius $R_{\mathrm{CH}}$ are selected as CHs. Three CH-separation settings were evaluated by setting $R_{\mathrm{CH}}$ to $R_{s}$, $1.2R_{s}$, and $R_{\mathrm{comm}}$, respectively. Non-CH sensors associate with CHs through the region-based membership mechanism in Section 3.2. Joining depends on local node degree, node–CH distance, activator values, and normalized residual energy.

The three $R_{\mathrm{CH}}$ settings evaluated the effect of explicit CH separation in the implemented protocol. No energy-dependent wavelength relationship was assumed.

Activity within each cluster was determined by local membership and sleep-state control. A node contributed to A(t) only if it was alive, not sleeping, and either selected as a CH or accepted as a member. Sleeping and dead nodes were excluded from sensing, control-message exchange, data transmission, coverage, and redundancy evaluation in that round. After cluster membership decisions, rejected and unclustered non-CH nodes entered sleep mode until the next scheduled clustering epoch.

EARDC was compared with four clustering protocols. All baselines were reimplemented in the same simulation environment to use a common deployment process, radio energy model, coverage grid, lifetime definition, and data transmission assumption. LEACH used probabilistic CH rotation, and HEED used residual energy and intra-cluster communication cost in distributed CH selection. WIFN [55] was included as an optimization-assisted clustering reference with stronger base-station involvement and global network information. Centralized coverage schedulers were not used as primary baselines because they directly optimize the active sensing set or coverage map, which differs from the clustering protocol comparison considered here.

The C3 implementation was adjusted for this comparison because the original protocol combines clustering, redundancy identification, gateway-assisted forwarding, and coverage evaluation. It was included because it also avoids exact coordinate requirements and organizes the network using RSSI-derived virtual rings, CH alternation, and redundancy identification. To isolate clustering and active sensing behavior, gateway-assisted forwarding was disabled, and CHs transmitted directly to the base station under the same data transmission model used by the other protocols. This preserved the C3 clustering and redundancy-identification mechanisms and removed a routing layer that was outside the scope of the present evaluation. In this reimplementation, every node counted as covered by sensing had to also deliver its data to a CH, which kept coverage and data collection consistent for all protocols. The results should therefore be interpreted as a clustering-focused C3 comparison rather than as a reproduction of the full gateway-based C3 system. Each configuration was evaluated over 300 independent simulation runs.

The inner joining distance in Table 5 was set to $0.8R_{s}$ because two equal sensing disks separated by $0.8R_{s}$ overlap by approximately 50%. This value defines a balanced boundary between the high overlap inner region and the lower overlap outer region. The additional too-close exclusion at $0.2R_{s}$ is stricter and corresponds to approximately 87% overlap. It was used only to prevent nearly duplicate sensing immediately next to an active CH when other alive neighbors were available.

### 6.2. Computational Complexity and Communication Overhead

The computational complexity follows the local operations in Algorithms 1 and 2. Let $N_{\mathrm{alive}}=\left|V\left(t\right)\right|$ denote the number of alive nodes, $M_{\mathrm{alive}}=\left|E\left(t\right)\right|$ the number of undirected links between alive one-hop neighbors, $\delta_{i}=\left|N_{i}\right|$ the one-hop degree of node *i*, and $\delta_{\max}=\max_{i}\delta_{i}$ the maximum one-hop node degree. At a clustering epoch, node *i* processes activator and inhibitor values from its $\delta_{i}$ one-hop neighbors for the graph-based reaction–diffusion update. The local-maximum comparison used for CH selection and the evaluation of advertised CHs also require $O(\delta_{i})$ operations. Local reaction and membership-probability calculations require constant time and do not change this order. A CH processes the join requests received from its one-hop neighbors. Since $\sum_{i}\delta_{i}=2M_{\mathrm{alive}}$ and each non-CH node sends at most one join request, the aggregate computational complexity of a clustering epoch is

$$O\;\left(N_{\mathrm{alive}}+M_{\mathrm{alive}}\right).$$

The worst-case computation at an individual node is $O(\delta_{\max})$. Between clustering epochs, each active member performs constant-time sensing and data transmission operations per round. A CH processes one packet from each active member assigned to its cluster. Since each member belongs to at most one cluster, the aggregate computation per round is $O(N_{\mathrm{alive}})$. The grid-based calculations used to measure coverage, overlap, and redundancy are simulation evaluation procedures and are not computations performed by sensor nodes as part of EARDC.

Cluster-formation control overhead is incurred at clustering epochs. Each alive node broadcasts one 80-bit activator–inhibitor message to its one-hop neighbors for the reaction–diffusion update. After this update, each alive node broadcasts one 48-bit activator message for the local-maximum comparison. Let $N_{\mathrm{CH}}=\left|H\left(t\right)\right|$ denote the number of selected CHs, $N_{\mathrm{join}}$ the number of join requests, and $N_{\mathrm{rej}}$ the number of rejection notifications at a clustering epoch. Each CH sends one 128-bit announcement, each non-CH node sends at most one 128-bit join request, and each node rejected during membership control receives one 128-bit rejection notification. The transmitted control traffic is therefore

$$\begin{array}{cc} B_{\mathrm{TX}} & =80N_{\mathrm{alive}}+48N_{\mathrm{alive}}+128\left(N_{\mathrm{CH}}+N_{\mathrm{join}}+N_{\mathrm{rej}}\right)\\ & =128\left(N_{\mathrm{alive}}+N_{\mathrm{CH}}+N_{\mathrm{join}}+N_{\mathrm{rej}}\right)\;\mathrm{bits}\;\mathrm{per}\;\mathrm{clustering}\;\mathrm{epoch}. \end{array}$$

Each activator–inhibitor and activator broadcast is received by all one-hop neighbors. Using $\sum_{i}\delta_{i}=2M_{\mathrm{alive}}$, the corresponding received control traffic is

$$B_{\mathrm{RX}}=256M_{\mathrm{alive}}+128\left(\sum_{c\in H(t)}\delta_{c}+N_{\mathrm{join}}+N_{\mathrm{rej}}\right)\;\mathrm{bits},$$

where $\delta_{c}$ is the one-hop degree of CH *c*. The first term accounts for reception of the 80-bit activator–inhibitor and 48-bit activator broadcasts. The remaining terms account for CH announcements, join requests, and rejection notifications. Since $N_{\mathrm{CH}}\leq N_{\mathrm{alive}}$, $N_{\mathrm{join}}\leq N_{\mathrm{alive}}$, and $N_{\mathrm{rej}}\leq N_{\mathrm{alive}}$, the number of control transmissions is $O(N_{\mathrm{alive}})$ and the number of control-message receptions is $O(N_{\mathrm{alive}}+M_{\mathrm{alive}})$ per clustering epoch.

These control messages are exchanged only at scheduled clustering epochs, which occur once every $T_{\mathrm{epoch}}$ rounds. No cluster-formation control messages are exchanged in the intermediate rounds. The stated transmission and reception complexities apply to one clustering epoch and include only clustering control messages. The energy model also includes the per-round sensing and data transmission costs of the active sensing set.

The energy cost of this communication overhead is obtained from the same first-order radio model used for data transmission. For a message of *k* bits transmitted over distance $d\leq R_{\mathrm{comm}}$, the transmission and reception costs are

(50) $$\begin{array}{cc} E_{\mathrm{TX}}\left(k,d\right) & =kE_{\mathrm{elec}}+k\epsilon_{\mathrm{fs}}d^{2},\\ E_{\mathrm{RX}}\left(k\right) & =kE_{\mathrm{elec}}, \end{array}$$

where $E_{\mathrm{elec}}$ is the transmitter and receiver electronics energy, and $\epsilon_{\mathrm{fs}}$ is the transmit amplifier energy listed in Table 5. At each clustering epoch, the simulator charges one transmission cost at $R_{\mathrm{comm}}$ for each 80-bit activator–inhibitor broadcast and each 48-bit activator broadcast, together with a reception cost at every one-hop neighbor. CH announcements are also charged at $R_{\mathrm{comm}}$. Join requests and rejection notifications are charged as 128-bit unicast messages using their node–CH transmission distances. Their sum defines $E_{\mathrm{ctrl}}$, which is deducted from node residual energy at that clustering epoch.

### 6.3. Results

The evaluation covers energy depletion, sensing performance, CH separation, and the contribution of the EARDC participation-control procedure. FND and network lifetime characterize the first and last node energy-depletion events. Time-series and lifetime-averaged metrics describe coverage, active participation, overlap, redundancy, and variation in the number of clusters. Coverage retention and coverage-bounded lifetime measure coverage service at specified thresholds. The $R_{\mathrm{CH}}$ sensitivity analysis, run-level statistical tests, and ERD-only ablation evaluate CH separation, differences among protocols, and the joint contribution of membership and sleep-state control. These measures are considered together because network lifetime, area coverage, and sensing redundancy are distinct performance objectives.

#### 6.3.1. Network Lifetime

The lifetime comparison used two indicators: FND and network lifetime. FND indicates when the first sensor exhausts its energy, and network lifetime measures the time until the last node dies.

Under the setup in Section 6.1, EARDC gave the longest average network lifetime among the evaluated protocols, with larger differences at higher deployment density. At λ=0.001, EARDC ($R_{\mathrm{CH}}\geq 1.2R_{s}$) reached 5551.7 rounds, compared with 5098.6 rounds for C3. At λ=0.005, EARDC ($R_{\mathrm{CH}}\geq R_{s}$) reached 11,419.0 rounds, compared with 8319.9 rounds for C3. At λ=0.02, EARDC ($R_{\mathrm{CH}}\geq R_{\mathrm{comm}}$) reached 16,327.4 rounds, compared with 6565.8 rounds for C3.

WIFN recorded the largest FND at all three densities, with average values of 2422.1, 2758.4, and 2881.7 rounds under its optimization-assisted CH selection and global-information assumptions. The main lifetime advantage of EARDC appeared in the last node death rather than FND. Compared with C3, the EARDC variants had higher FND at λ=0.001, lower FND at λ=0.005, and higher FND at λ=0.02. At the highest density, EARDC ($R_{\mathrm{CH}}\geq R_{\mathrm{comm}}$) reached 1784.5 rounds compared with 1299.6 rounds for C3.

The three EARDC variants showed how $R_{\mathrm{CH}}$ affected network lifetime. At λ=0.001, the three settings gave similar lifetimes, which indicates a limited effect of CH separation in the sparse deployment. At λ=0.005, the $R_{\mathrm{CH}}\geq R_{s}$ setting gave the longest EARDC lifetime, although the three variants remained relatively close. At λ=0.02, EARDC ($R_{\mathrm{CH}}\geq R_{\mathrm{comm}}$) gave the longest lifetime. The preferred setting therefore depends on deployment density and the performance measure considered.

**Figure 7:**
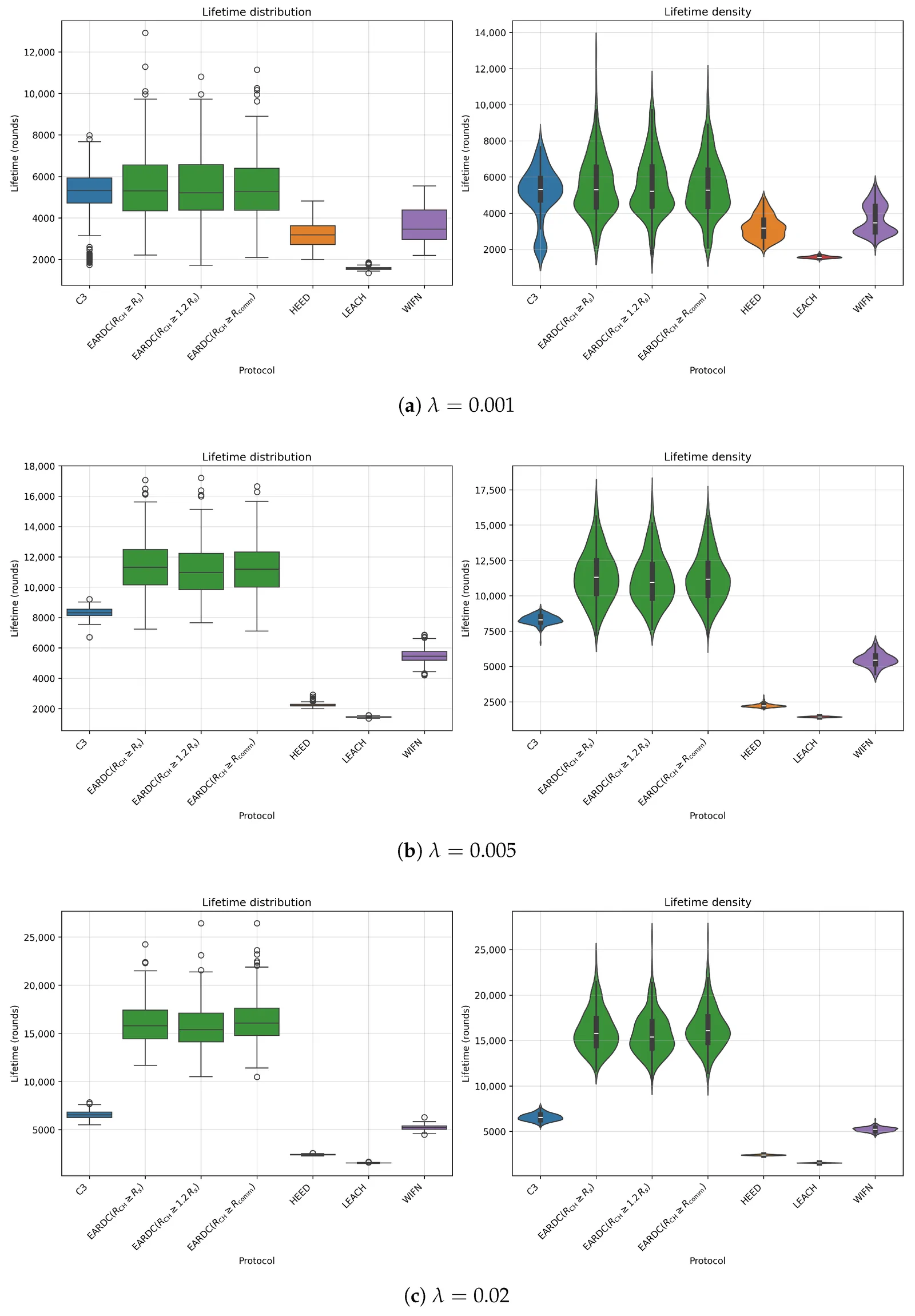
Network lifetime distributions for C3, EARDC, HEED, LEACH, and WIFN under the three deployment densities. Network lifetime denotes the last node death.

**Figure 8:**
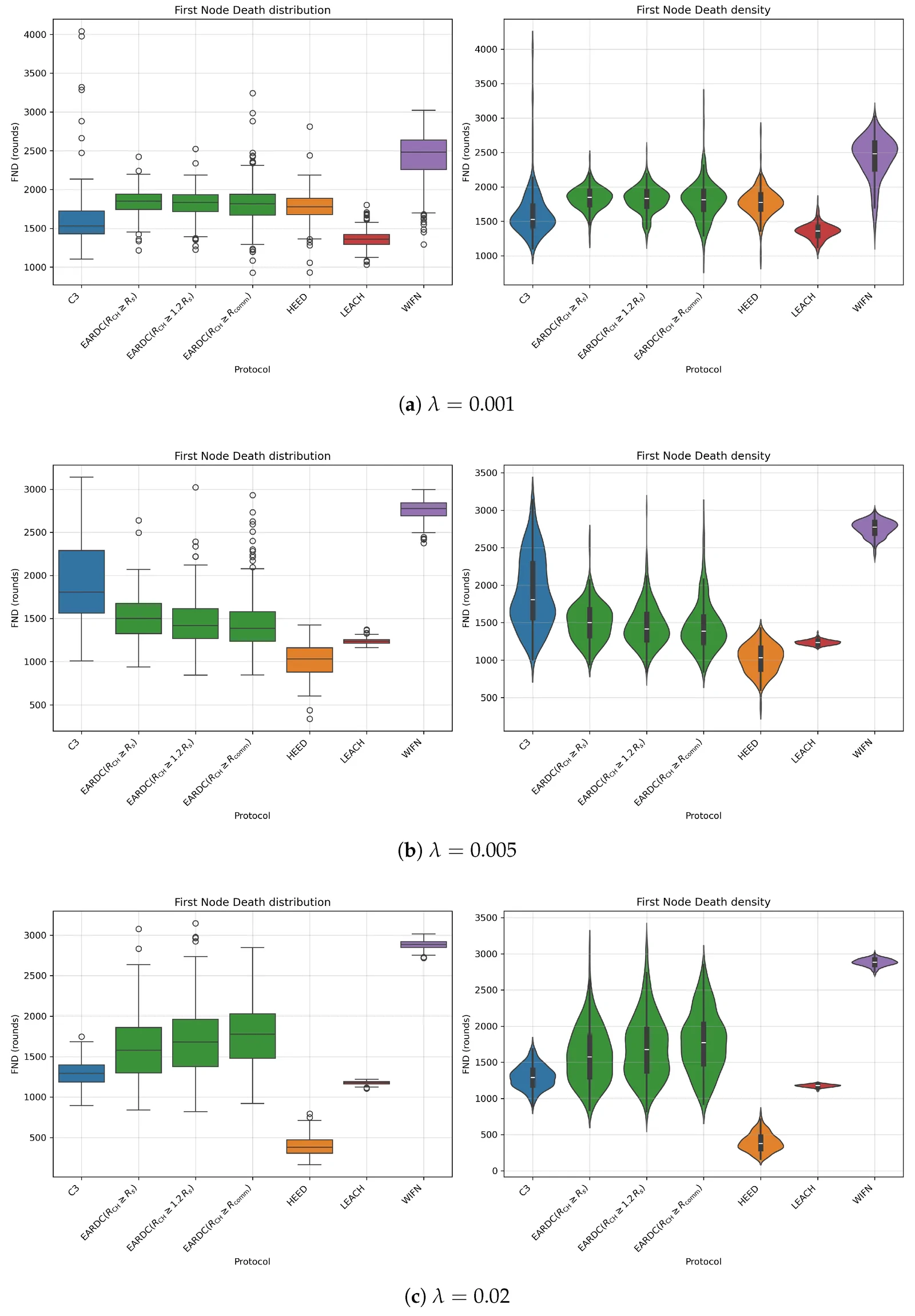
First node death distributions for C3, EARDC, HEED, LEACH, and WIFN under the three deployment densities. FND denotes the first node death.

Figure 7 and Figure 8 summarize the distributions of network lifetime and FND over simulation runs for the three deployment densities.

#### 6.3.2. Coverage and Redundancy Metrics

In addition to FND and network lifetime, the protocols were compared using coverage, active-node ratio, alive node ratio, number of clusters, overlap ratio, redundancy degree, redundant node ratio, and cluster stability. These metrics were averaged over each protocol’s lifetime. Coverage, overlap, and redundancy were computed from the active sensing set, and the active-node ratio was the simulation measure corresponding to the analytical active-node density $\lambda_{\mathrm{act}}\left(x,t\right)$. Lower redundancy degree and lower cluster stability values indicate less duplicate sensing and a more stable number of clusters, respectively.

**Figure 9:**
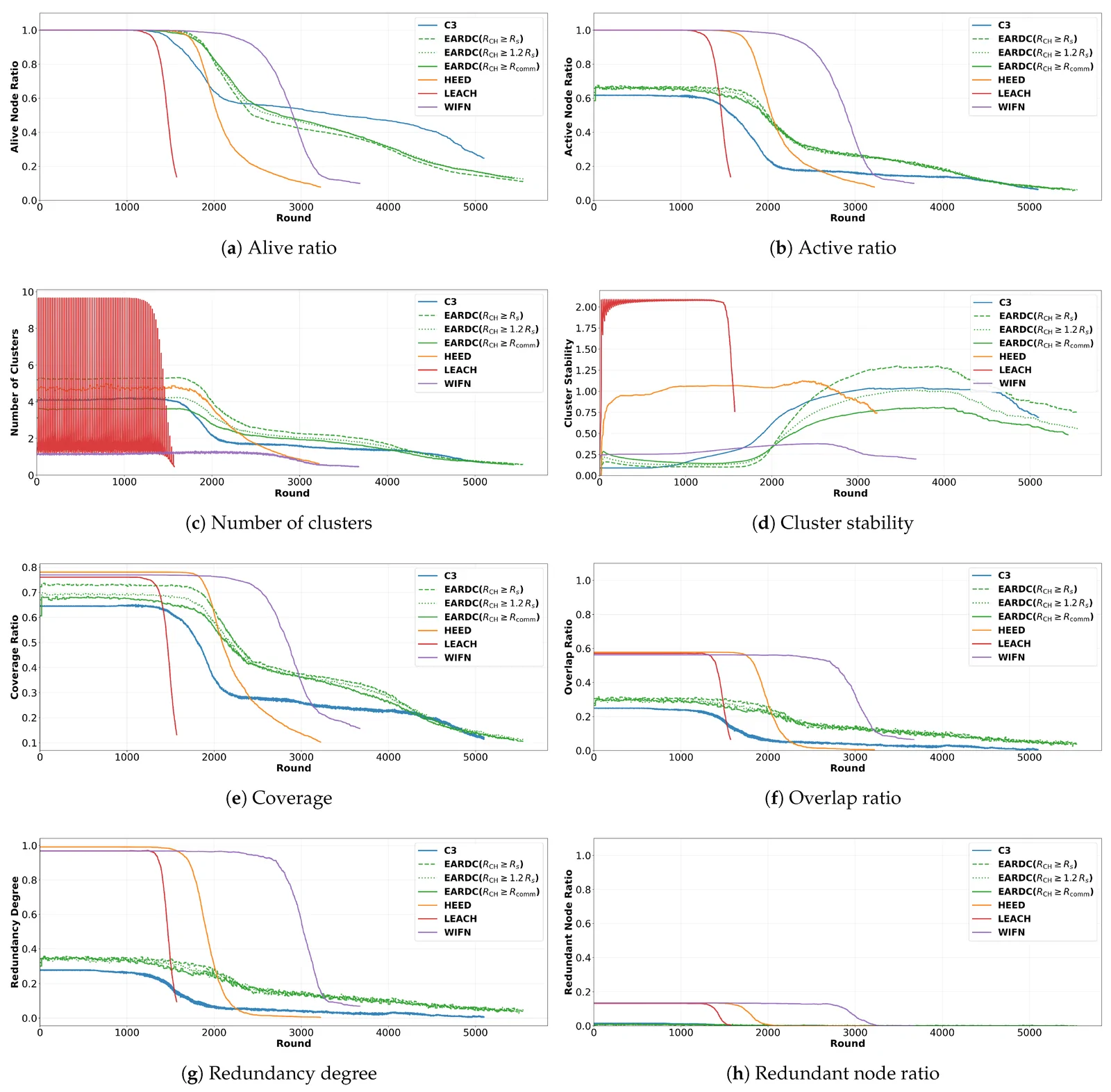
Time-series performance metrics at deployment density λ=0.001 nodes/$m^{2}$. Each curve gives the per-round average over 300 simulation runs. After the last node dies in a run, that run contributes a metric value of zero in subsequent rounds.

**Figure 10:**
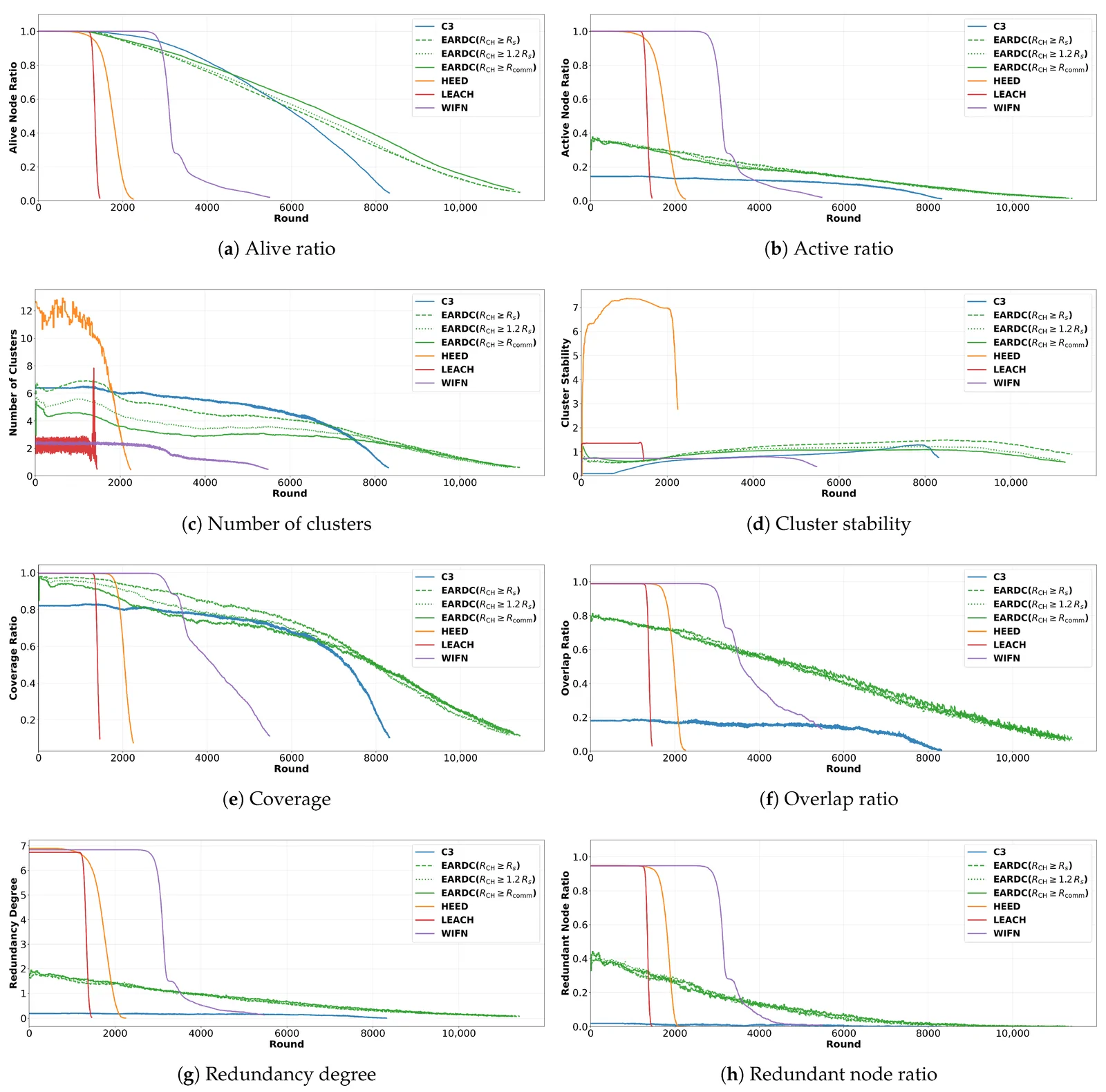
Time-series performance metrics at deployment density λ=0.005 nodes/$m^{2}$. Each curve gives the per-round average over 300 simulation runs. After the last node dies in a run, that run contributes a metric value of zero in subsequent rounds.

**Figure 11:**
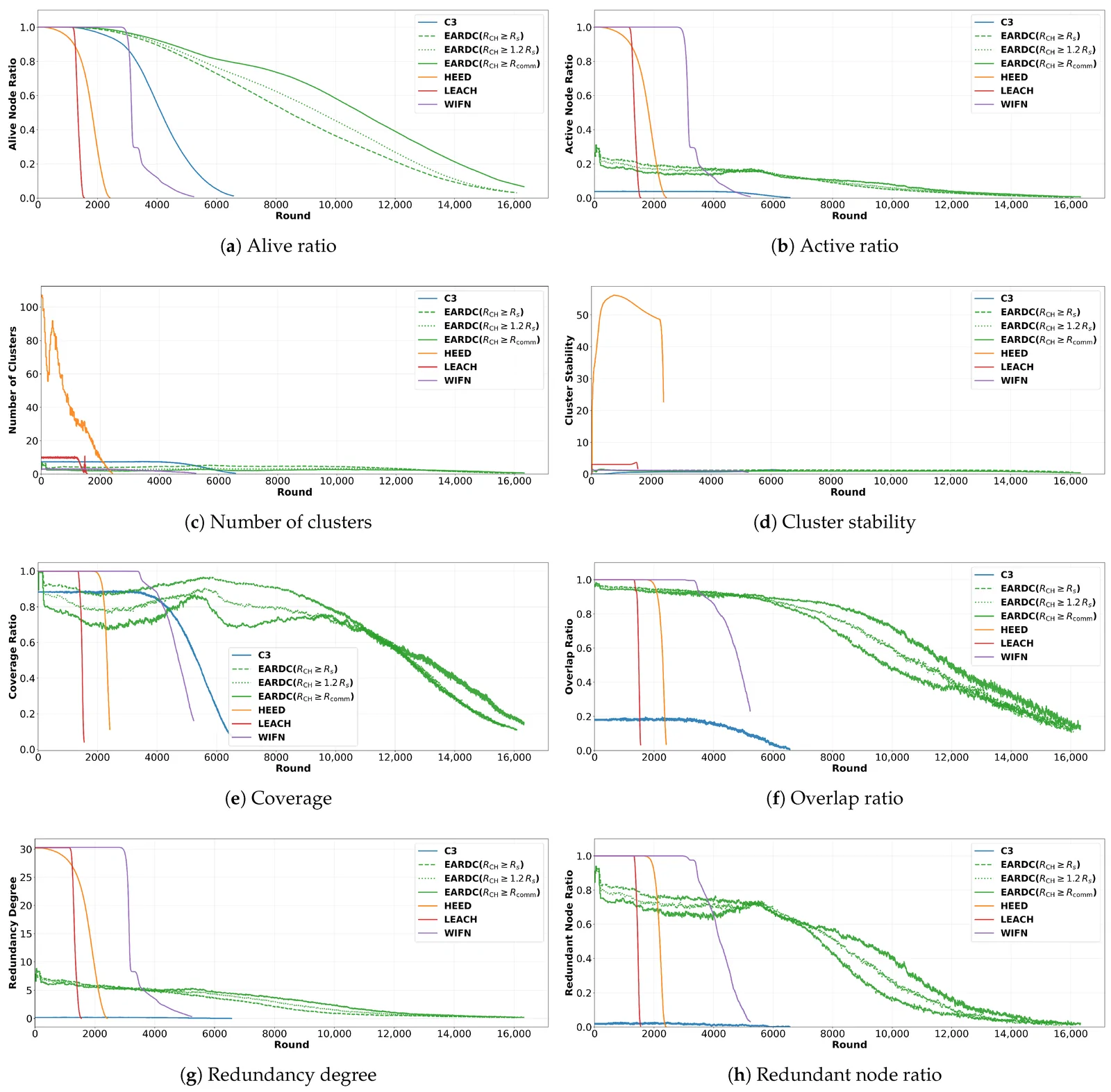
Time-series performance metrics at deployment density λ=0.02 nodes/$m^{2}$. Each curve gives the per-round average over 300 simulation runs. After the last node dies in a run, that run contributes a metric value of zero in subsequent rounds.

The lifetime averages in Table 6, Table 7 and Table 8 should be interpreted with the corresponding time-series plots. A longer lifetime includes more late rounds with lower coverage or activity and can therefore reduce a lifetime-averaged value. Figure 9, Figure 10 and Figure 11 show how coverage, redundancy, and cluster formation evolve until the last node exhausts its energy.

The time-series plots show the per-round behavior before the lifetime averages are considered. At λ=0.001, the EARDC and C3 trajectories are relatively close because the sparse deployment provides few alternatives for selective sensing participation. At λ=0.005, EARDC operates longer than C3 and has higher overlap and redundancy. At λ=0.02, EARDC maintains the alive node ratio and coverage for more rounds than C3. Its active ratio and redundancy remain below those of HEED, LEACH, and WIFN. Its variation in the number of clusters is also substantially lower than that of HEED and LEACH and comparable to that of WIFN.

The trajectories of the number of clusters and cluster stability show temporal variation in the CH population. EARDC and C3 maintain relatively stable numbers of clusters, although their average numbers differ. WIFN generally maintains a small and stable number of clusters. LEACH exhibits periodic changes in the number of clusters in the sparse deployment, and HEED shows a large initial CH population followed by a rapid decrease in the dense deployment.

The LEACH and HEED trajectories for the number of clusters reflect their CH election mechanisms under the evaluated densities. The LEACH variation at λ=0.001 follows from the LEACH epoch threshold with p=0.05. With about ten nodes on average in the sparse deployment, the threshold can increase late in an epoch and select a large fraction of the remaining alive nodes as CHs in the same round. For HEED at λ=0.02, many nodes satisfy the local CH election conditions during the initial rounds. As the number of alive nodes decreases over HEED’s shorter lifetime, the number of CHs drops rapidly.

Table 6, Table 7 and Table 8 summarize the corresponding metrics averaged over lifetime. At low density, all protocols are constrained by limited spatial redundancy. EARDC gives a moderate lifetime improvement over C3 with comparable coverage. HEED, LEACH, and WIFN achieve higher coverage with larger active ratios and redundancy.

At higher deployment densities, the lifetime averages show the tradeoff observed in the time-series plots. C3 has higher lifetime-averaged coverage and lower overlap and redundancy than EARDC. Its network lifetime is shorter. HEED and LEACH also achieve high lifetime-averaged coverage at λ=0.02, although those values are obtained with high active ratios and short lifetimes. EARDC uses a smaller active fraction than HEED, LEACH, and WIFN and maintains moderate to high coverage over a longer network lifetime.

The active ratio values show that EARDC limits simultaneous participation as deployment density increases. For the $R_{\mathrm{CH}}\geq R_{\mathrm{comm}}$ configuration, the active ratio decreases from 0.363±0.220 at λ=0.001 to 0.159±0.098 at λ=0.005 and 0.096±0.059 at λ=0.02. This decrease indicates that EARDC does not activate nodes in proportion to deployment density, even though the number of active nodes can increase in denser deployments.

The redundancy values further clarify this behavior. C3 maintains very low redundancy degree and smaller overlap than EARDC. At λ=0.005, EARDC has higher redundancy degree than C3, with values between 0.734 and 0.785, and remains well below HEED, LEACH, and WIFN. EARDC redundancy is higher at λ=0.02, where the absolute number of active nodes and accepted members is also larger. For $R_{\mathrm{CH}}\geq R_{\mathrm{comm}}$, the redundancy degree is 3.250, compared with 21.998, 25.997, and 19.458 for HEED, LEACH, and WIFN, respectively.

The cluster stability values quantify the variation in the number of CHs shown in the time-series plots. At λ=0.005, the EARDC values remain between 0.933 and 1.144, compared with 6.769 for HEED. At λ=0.02, the EARDC values remain between 0.954 and 1.254, compared with 50.323 for HEED and 3.108 for LEACH. C3 has less variation in the number of clusters than EARDC. Its network lifetime is much shorter at high density.

Last node death identifies the energy-depletion endpoint of a simulation run. It does not establish how long an area-coverage requirement is satisfied. Table 9 and Table 10 therefore extend the comparison to the five thresholds θ∈{0.9,0.8,0.7,0.6,0.5} for all seven configurations. Both metrics were calculated for each simulation run and then averaged over the 300 runs. Coverage retention $T_{\mathrm{cov}}\left(\theta \right)$ is the number of consecutive rounds from network initialization until the coverage ratio first falls below θ. Coverage-bounded lifetime $L_{\mathrm{cov}}\left(\theta \right)$ counts the cumulative number of rounds that satisfy the threshold. The second measure was included because the active sensing set was recomputed at scheduled clustering epochs. Coverage can fall below a threshold and later recover. The first violation does not necessarily mark permanent loss of coverage service.

At λ=0.001 and 0.005, EARDC with $R_{\mathrm{CH}}\geq R_{s}$ exceeds C3 in both coverage retention and coverage-bounded lifetime at every evaluated threshold. The dense deployment presents a different tradeoff. EARDC with $R_{\mathrm{CH}}\geq R_{\mathrm{comm}}$ has the longest last-node-death lifetime. Its lifetime-averaged coverage is 0.635, compared with 0.729 for C3. Each protocol’s lifetime-averaged coverage was calculated from network initialization to its own last node death. Because EARDC operated longer than C3, the EARDC average included later rounds after C3 reached its last node death. The two values therefore summarize coverage over different durations. Its coverage retention is also shorter than that of C3 at all five thresholds. For example, at θ=0.7, the average retention times are 829 rounds for this EARDC configuration and 4224 rounds for C3.

Coverage-bounded lifetime gives the complementary service-duration result. In the dense deployment, EARDC with $R_{\mathrm{CH}}\geq R_{\mathrm{comm}}$ has a larger coverage-bounded lifetime than C3 at every threshold. At θ=0.7, the corresponding values are 7884 and 4750 rounds. EARDC reconstructs the active sensing set at scheduled clustering epochs. Coverage can therefore rise above the threshold again after an earlier violation, and these later qualifying rounds contribute to coverage-bounded lifetime. EARDC with $R_{\mathrm{CH}}\geq R_{s}$ has the largest average coverage-bounded lifetime among the seven configurations at all five thresholds in the dense deployment. The two threshold-based metrics therefore distinguish continuous coverage retention from the cumulative duration of useful coverage and prevent last node death from being interpreted as coverage service lifetime.

#### 6.3.3. Analytical Model Evaluation

The analytical approximations for active-node density and coverage were compared with run-level simulation measurements for the three deployment densities and three CH separation settings. Only clustering epochs before first node death (FND) were included. The analytical expressions used the initial deployment density, which no longer described the alive node density after node depletion had begun. For each setting, a fixed run-identifier split assigned 150 runs to estimation and 150 runs to validation. Each run contributed one observation averaged over the included clustering epochs.

A Matérn Type II hard-core process was also evaluated as a diagnostic reference for the separation imposed by $R_{\mathrm{CH}}$ [56]. For a parent PPP with density λ, the hard-core density is

(51) $$\lambda_{\mathrm{MII}}=\frac{1-\exp(-\lambda \pi R_{\mathrm{CH}}^{2})}{\pi R_{\mathrm{CH}}^{2}}.$$

This reference accounts for the exclusion distance. It does not include the activator values, residual energy, one-hop connectivity, or finite deployment boundary that affect EARDC CH selection. Table 11 compares it with the simulated CH density. It was not used to calculate active-node density or coverage.

Calculation of active-node density also requires $q_{\mathrm{MS}}$, the conditional probability that an alive non-CH node belongs to the active sensing set after membership acceptance and temporary sleep-state control. For each estimation run, $q_{\mathrm{MS}}$ was estimated as the ratio of active non-CH node observations to alive non-CH node observations over the included clustering epochs. Table 12 reports the unweighted mean of these run-level estimates. Validation runs were not used for estimation.

For the active-node density comparison, the CH probability was obtained from the validation-run CH density and combined with $q_{\mathrm{MS}}$ estimated from the independent estimation runs. This conditional calculation evaluated the aggregate active-probability expression without using the Matérn reference as an EARDC predictor. Area coverage was then calculated from the Poisson point-coverage expression for the finite monitoring region. Numerical integration at the simulation grid-cell centers accounted for sensing disks truncated by the region boundary. The PPP assumption applies only to the initial sensor deployment; it does not describe the CH or active-node locations produced by EARDC. The coverage calculation was therefore based on aggregate active-node density. The overlap and redundancy expressions were not evaluated separately. The absolute percentage difference from the Matérn reference was $|e_{\mathrm{MII}}\left|=100\right|\lambda_{\mathrm{CH}}^{\mathrm{sim}}-\lambda_{\mathrm{MII}}|/\lambda_{\mathrm{MII}}$. For the conditional active-node density and coverage estimates, the absolute percentage error was $100|{\bar{x}}_{\mathrm{val}}-x_{\mathrm{est}}|/x_{\mathrm{est}}$, where ${\bar{x}}_{\mathrm{val}}$ is the validation-run mean, and $x_{\mathrm{est}}$ is the corresponding conditional estimate.

The Matérn reference and simulated CH density both decrease as $R_{\mathrm{CH}}$ increases. Differences of 2.3–72.3% show that the reference is unsuitable for quantitative prediction of EARDC CH density.

The estimated values of $q_{\mathrm{MS}}$ range from 0.176 to 0.406 and vary with deployment density and $R_{\mathrm{CH}}$. The conditional non-CH participation probability is therefore specific to each deployment density and CH separation setting. When the aggregate active-probability expression is conditioned on the simulated CH density, the active-node density differences range from 0.6% to 6.3%. This comparison evaluates whether active-node density can be estimated from the simulated CH density and the independently estimated non-CH participation probability. It does not predict which individual nodes become CHs or active members.

Table 13 compares the conditional Poisson coverage estimates with simulated area coverage. The absolute differences between the conditional finite-area Poisson calculation and simulated coverage range from 2.7% to 22.1%. The calculation underestimates coverage at λ=0.001 and 0.005. It overestimates coverage at λ=0.02. Both the simulated and conditional values decrease as $R_{\mathrm{CH}}$ increases within each density. The calculation does not reproduce the coverage response between deployment densities. Simulated coverage at λ=0.02 is lower than at λ=0.005, although the Poisson calculation increases with active-node density. The discrepancy reflects the dependence introduced by CH separation, membership acceptance, and sleep-state control. Accordingly, the analytical expressions characterize aggregate relationships and their limitations. They are not presented as independently validated predictors of EARDC CH density or coverage.

#### 6.3.4. Sensitivity and Statistical Significance Analysis

**Figure 12:**
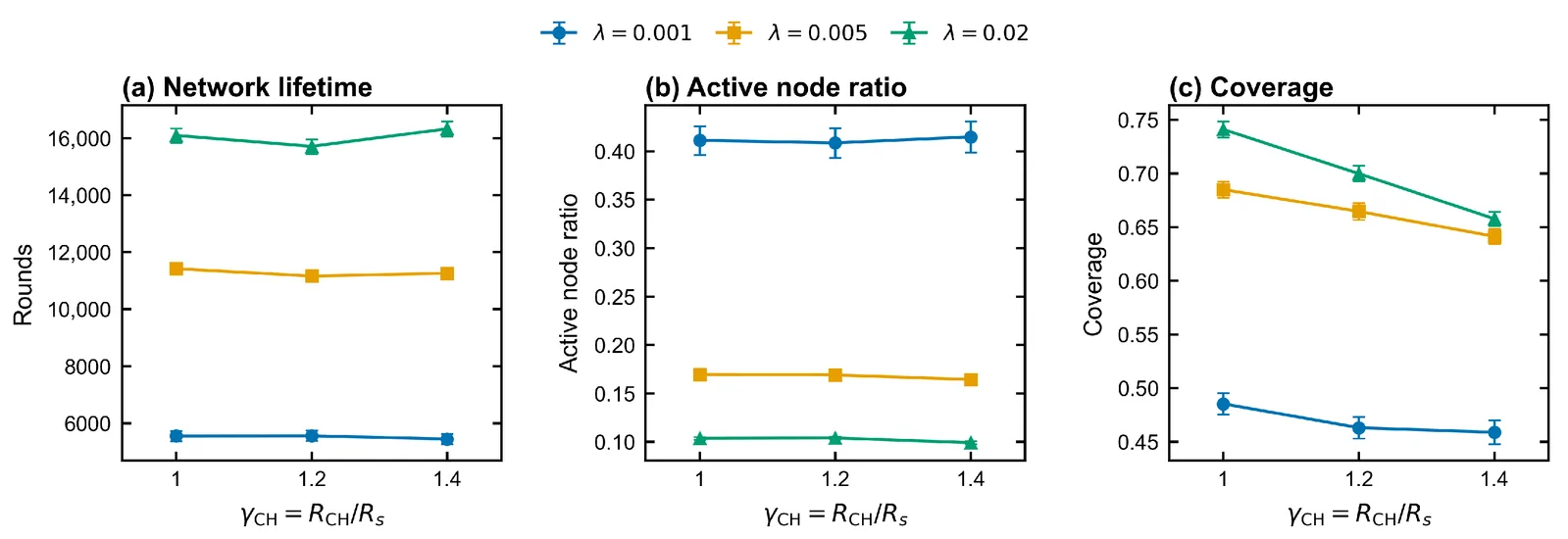
Sensitivity of EARDC to the normalized CH separation radius $\gamma_{\mathrm{CH}}=R_{\mathrm{CH}}/R_{s}$ at the three deployment densities. Markers show averages over 300 independent simulation runs, and error bars show 95% confidence intervals.

The sensitivity of EARDC to the CH separation radius was evaluated using $\gamma_{\mathrm{CH}}=R_{\mathrm{CH}}/R_{s}\in \left\{1,1.2,1.4\right\}$. These values correspond to $R_{s}$, $1.2R_{s}$, and $R_{\mathrm{comm}}$. Figure 12 shows the average network lifetime, run-level average active-node ratio, and run-level average coverage. Error bars indicate 95% confidence intervals from 300 independent runs for each setting. The run-level summaries assign equal weight to each simulation realization; Table 6, Table 7 and Table 8 instead summarize the per-round trajectories over each configuration’s average lifetime.

Network lifetime does not change monotonically with $\gamma_{\mathrm{CH}}$ for all three deployment densities. The differences are small in the sparse deployment. The smallest evaluated radius gives the longest lifetime at λ=0.005, and the largest radius gives the longest lifetime at λ=0.02. The active-node ratio changes less than coverage over the evaluated range. Coverage decreases as $\gamma_{\mathrm{CH}}$ increases at each density. This behavior is consistent with the lower number of CHs in Table 6, Table 7 and Table 8 under a larger separation radius. The results show a density-dependent tradeoff among CH availability, active participation, coverage, and network lifetime. No evaluated value of $R_{\mathrm{CH}}$ gives the best result for every metric and density.

The dense-deployment results show this tradeoff directly. As $R_{\mathrm{CH}}$ increases from $R_{s}$ to $1.2R_{s}$ and $R_{\mathrm{comm}}$, the average number of CHs decreases from 3.817 to 2.842 and 2.297. Average coverage decreases from 0.721 to 0.679 and 0.635. The corresponding network lifetimes are 16,094, 15,712, and 16,327 rounds. A larger separation radius reduces the number of simultaneous CHs and can lower the active-node ratio. It can also increase the sensing and communication load assigned to the remaining CHs and members. These effects act in different directions and produce the nonmonotonic lifetime result. The selection of $R_{\mathrm{CH}}$ therefore depends on the required balance between coverage and lifetime. In the evaluated dense deployment, $R_{s}$ provides the highest average coverage among the EARDC settings, and $R_{\mathrm{comm}}$ provides the longest network lifetime.

Run-level statistical tests were performed separately for each deployment density to test equality among the configuration means. Each configuration contributed 300 independently generated simulation runs. For each time-varying metric, one lifetime average was calculated per run before statistical testing. The FND analysis at λ=0.001 contained 2097 valid observations because three runs did not produce valid FND values. For each metric and density, the observed Welch ANOVA statistic was compared with a distribution obtained from 999 random permutations of the configuration labels. For every metric summarized in Table 6, Table 7 and Table 8, the omnibus tests gave $p_{\mathrm{perm}}=0.001$ at all three deployment densities. This is the smallest *p*-value resolvable with 999 permutations. Kruskal–Wallis tests also rejected equality among the configurations for these metrics.

Rejection of an omnibus null hypothesis indicates that at least one configuration differs but does not identify the individual differences. Pairwise comparisons therefore used the absolute Welch *t* statistic with 999 random permutations of the configuration labels within each pooled pair. For each metric reported in Table 14, Holm correction was applied separately to the 21 pairwise comparisons among the seven configurations at each deployment density. Table 14 summarizes the adjusted *p*-values for EARDC with $R_{\mathrm{CH}}=R_{\mathrm{comm}}$ compared with C3, HEED, LEACH, and WIFN. The adjusted *p*-values for network lifetime, active ratio, and coverage range from 0.021 to 0.032 and remain below the 0.05 significance level at every density. The magnitude and direction of these differences are reported in Table 15.

Statistical significance alone does not describe the magnitude or practical importance of a difference. Table 15 therefore reports the mean difference $\Delta ={\bar{x}}_{\mathrm{EARDC}}-{\bar{x}}_{\mathrm{baseline}}$ and its 95% confidence interval for the principal lifetime, participation, and coverage outcomes. The intervals use the Welch standard error and Welch–Satterthwaite degrees of freedom. They provide the primary pairwise magnitude estimates under unequal variances. Descriptive omnibus effect sizes from the one-way ANOVA are also reported. The $\eta^{2}$ values for network lifetime, active ratio, and coverage are 0.559, 0.743, and 0.537 at λ=0.001; 0.919, 0.991, and 0.828 at λ=0.005; and 0.945, 0.998, and 0.861 at λ=0.02, respectively.

The effect estimates confirm substantive differences whose directions depend on the metric and baseline. Compared with C3, EARDC has a longer network lifetime at every density. It has a larger active ratio at every density and lower run-level average coverage at λ=0.005 and 0.02. Compared with HEED, LEACH, and WIFN, EARDC has a longer network lifetime and a lower active ratio at all three densities. Its run-level average coverage is lower. These comparisons quantify the tradeoff among network lifetime, coverage, and active ratio and show that no protocol provides the most favorable result for every metric.

#### 6.3.5. Effect of Membership and Sensing Participation Control in EARDC

To isolate the contribution of membership and sensing participation control, EARDC was compared with an energy-aware reaction–diffusion-only (ERD-only) configuration. The ERD-only configuration maintained the residual-energy scaled activator–inhibitor update and local CH selection. It omitted the membership and temporary sleep-state control used to construct the EARDC active sensing set. Both configurations used the fixed CH separation radius $R_{\mathrm{CH}}=R_{\mathrm{comm}}$ at all three deployment densities, so their differences were evaluated under the same CH separation setting.

In EARDC, the membership decision determines the sleep state of each non-CH node. Accepted non-CH nodes remain active members, and rejected or otherwise unclustered non-CH nodes sleep until the next scheduled clustering epoch. ERD-only excludes both membership decisions and sleep-state control. The performance differences between the two configurations therefore reflect the combined effects of EARDC membership decisions and the resulting sleep states. Separate evaluation of these effects would require an additional protocol variant in which rejected or otherwise unclustered non-CH nodes do not automatically enter the sleep state. Such a variant was not evaluated in this study.

Table 16 shows that EARDC has a longer network lifetime, a later FND, and a lower active ratio than ERD-only at all three deployment densities. The difference in network lifetime increases with deployment density. The fixed-radius comparison shows that residual-energy-aware CH selection alone does not explain the longer lifetime of EARDC. Membership acceptance and temporary sleep-state control reduce simultaneous sensing and data transmission. Compared with ERD-only, EARDC lowers the average active ratio by approximately 44%, 55%, and 52% at λ=0.001, 0.005, and 0.02, respectively.

Table 17 reports coverage retention $T_{\mathrm{cov}}\left(\theta \right)$ and coverage-bounded lifetime $L_{\mathrm{cov}}\left(\theta \right)$. At λ=0.001, ERD-only has higher values for both measures from θ=0.9 to 0.6 because its larger active set provides greater simultaneous sensing coverage. At θ=0.5, EARDC exceeds ERD-only on both measures because its longer network lifetime contributes more qualifying rounds. At λ=0.005 and 0.02, EARDC exceeds ERD-only in coverage retention and coverage-bounded lifetime at every evaluated threshold. The coverage effect of membership and sleep-state control is therefore density-dependent. The sparse-deployment result is consistent with the limited availability of alternative sensors after nodes are excluded from the active sensing set. At the two higher densities, more sensors remain available for participation in later clustering epochs.

### 6.4. Discussion

The simulations evaluated EARDC with graph-based activator–inhibitor updates, local CH selection, membership acceptance, and sleep-state control. Section 4 characterized the reaction–diffusion update under homogeneous residual energy. Section 5 estimated average active-node density from the CH-selection probability and conditional non-CH participation probability. The estimated active-node density was used to approximate coverage and redundancy. The simulations computed these metrics directly from the active sensing set. All protocols used the deployment process, radio energy model, coverage grid, lifetime definitions, and data transmission assumptions specified in Section 6.1.

The conditional active-node density calculation in Section 6.3.3 used simulated CH density and a conditional non-CH participation probability estimated from a separate set of runs. The subsequent Poisson coverage calculation followed the simulated decrease in coverage as $R_{\mathrm{CH}}$ increased within each density. However, it did not reproduce the simulated coverage differences among the three deployment densities. The aggregate analysis therefore describes selected network-level relationships but does not predict individual CH-selection, membership, or sleep state decisions.

The sensitivity results do not identify a single CH separation radius that performs best for all deployment densities and performance measures. Under the homogeneous PPP model, the expected number of deployed sensors within a CH competition disk is $\lambda \pi R_{\mathrm{CH}}^{2}$. At initial deployment, increasing $R_{\mathrm{CH}}$ therefore includes more sensors in each local CH comparison, and this increase is larger at higher density. In the simulations, a larger separation radius generally produced fewer CHs and lower coverage, but network lifetime did not change monotonically. The smallest evaluated radius gave the longest network lifetime at λ=0.005, and the largest radius gave the longest lifetime at λ=0.02. These results demonstrate a tradeoff among CH availability, coverage, active participation, and network lifetime. The sensitivity analysis is limited to the three evaluated CH separation settings and does not establish a universal parameter choice.

The threshold-based metrics distinguish last node death from coverage service. Coverage retention measures the duration before coverage first falls below a specified threshold. Coverage-bounded lifetime counts all rounds that meet the threshold, including qualifying rounds after coverage recovers following a scheduled clustering epoch. In the dense deployment, EARDC with $R_{\mathrm{CH}}=R_{\mathrm{comm}}$ had the longest last-node-death lifetime. Its coverage retention was lower than that of C3 at every evaluated threshold, but its coverage-bounded lifetime was higher. EARDC with $R_{\mathrm{CH}}=R_{s}$ had the largest coverage-bounded lifetime among the seven configurations at every threshold. These results show that last node death, coverage retention, and coverage-bounded lifetime measure distinct aspects of network service.

At all three deployment densities, EARDC had a higher active ratio than C3 and a lower active ratio than HEED, LEACH, and WIFN. C3 also had the lowest overlap and redundancy, but its network lifetime was shorter than that of EARDC. These results show that active ratio and sensing redundancy alone do not determine network lifetime. Energy consumption also depends on cluster roles because CHs receive and aggregate member data before transmitting to the base station. EARDC uses normalized residual energy in CH selection and reselects CHs at scheduled clustering epochs. Network lifetime must therefore be interpreted in relation to active participation and the energy costs assigned to CHs.

LEACH and HEED provide conventional clustering references. All alive nodes in these protocols continued to sense in the simulations. Dense deployments consequently produced high coverage with larger active ratios and redundancy. WIFN used global network information for CH selection and recorded the latest FND at all three densities. This result indicates stronger protection against early node energy depletion under the evaluated setup. EARDC had an earlier FND than WIFN but a longer last-node-death lifetime. Its smaller active sensing set allowed nonparticipating nodes to conserve energy for later clustering epochs. FND and last node death therefore characterize different stages of network energy depletion. These comparisons show that EARDC does not seek to maximize immediate sensing participation, delay the first node death, or minimize overlap in every round. It uses a selected active sensing set to maintain area coverage over a longer network lifetime.

For the seven configurations in the baseline comparison, the omnibus permutation tests rejected equality for every reported metric at each deployment density. This result indicates that at least one configuration differed and does not establish that one protocol performed best for every metric. The Holm-adjusted pairwise tests for network lifetime, active ratio, and coverage remained below the 0.05 level for EARDC with $R_{\mathrm{CH}}=R_{\mathrm{comm}}$ compared with C3, HEED, LEACH, and WIFN. The reported $\eta^{2}$ values quantify protocol-level variation, and the pairwise mean differences with 95% confidence intervals show the magnitude and direction of the principal lifetime, participation, and coverage differences. These estimates are considered with the metric values and time-series plots when assessing practical performance.

A separate ERD-only ablation compared EARDC with a configuration that used the same residual-energy-aware RD update and local CH selection but omitted membership control and temporary sleep states. With $R_{\mathrm{CH}}=R_{\mathrm{comm}}$ for both configurations, EARDC had a longer network lifetime, a later FND, and a lower active ratio at all three deployment densities. In EARDC, the sleep state of a non-CH node follows its membership decision. The comparison therefore evaluated membership and sleep-state control jointly and did not separate their individual contributions. At λ=0.001, ERD-only provided higher coverage retention and coverage-bounded lifetime from θ=0.9 to 0.6. At λ=0.005 and 0.02, EARDC exceeded ERD-only on both coverage measures at every evaluated threshold. The effect of membership and sleep-state control on coverage therefore depends on deployment density and the selected threshold. The ablation shows that residual-energy-aware CH selection alone does not explain the lifetime and coverage results of EARDC.

EARDC incurs computational and communication overhead at each clustering epoch. The aggregate computational complexity is $O(N_{\mathrm{alive}}+M_{\mathrm{alive}})$, where $M_{\mathrm{alive}}$ is the number of links between alive one-hop neighbors. Control-message exchange requires $O(N_{\mathrm{alive}})$ transmissions and $O(N_{\mathrm{alive}}+M_{\mathrm{alive}})$ receptions. These costs occur once every $T_{\mathrm{epoch}}$ rounds. The simulator deducts the corresponding transmission and reception energy from node residual energy. The reported lifetime results therefore include activator–inhibitor value exchange, activator broadcasts, CH announcements, join requests, and rejection notifications.

The network topology changes as nodes exhaust their energy, although sensor positions and communication radii remain fixed. A node with exhausted energy is excluded from sensing and data transmission immediately. At the next scheduled clustering epoch, alive nodes exchange activator and inhibitor values with their current one-hop neighbors. CH selection, membership, and the active sensing set are then recomputed. The simulations therefore evaluate topology changes caused by node energy depletion. They do not establish performance under node mobility, intermittent links, or abrupt node failures.

The comparative results are specific to homogeneous PPP deployments with fixed sensor positions, a centered base station, fixed sensing and communication radii, a square monitoring field, and direct CH-to-base-station transmission. Changing $R_{\mathrm{comm}}$ would alter one-hop neighbor sets, local activator–inhibitor value exchange, and cluster-joining opportunities. A different field size, deployment process, or base-station position would affect coverage geometry and CH-to-base-station transmission distances. The numerical results and protocol rankings should therefore not be assumed to remain unchanged under nonuniform deployment, other field geometries, different base-station locations, or different communication ranges. Such scenarios require a separate evaluation.

## 7. Conclusions and Future Work

This paper developed EARDC, a self-organizing energy-aware reaction–diffusion clustering framework with local sensing participation control for area coverage in wireless sensor networks. The objective was to maintain useful area coverage without requiring global topology information, exact node coordinates, or coverage computation at the base station. In EARDC, normalized residual energy scales the local activator–inhibitor update used for CH selection. Local membership decisions determine the temporary sleep states of non-CH nodes. Selected CHs and accepted members form the active sensing set.

The analysis examined the energy-aware reaction–diffusion update under homogeneous residual energy. Average active-node density was used to approximate coverage, overlap, and redundancy. The hexagonal model provided a geometric reference for the relationship among CH spacing, possible coverage gaps, and sensing overlap. These analytical results describe network-level relationships rather than individual CH locations or cluster memberships. Comparison with the validation runs showed smaller differences for active-node density than for finite-area coverage. Within each deployment density, the coverage calculation followed the simulated decrease associated with a larger $R_{\mathrm{CH}}$. However, it did not accurately estimate how coverage changed when deployment density changed. Because the calculation required simulated CH density and an estimated non-CH participation probability, it evaluated the relationship between average active-node density and coverage rather than independently predicting EARDC coverage.

The simulations showed a tradeoff among network lifetime, coverage service, active participation, and redundancy. EARDC had the longest last-node-death lifetime among the evaluated configurations at all three deployment densities. It also had a lower active fraction than LEACH, HEED, and WIFN. C3 provided stronger redundancy suppression. In the dense deployment, the lifetime-maximizing EARDC setting had lower coverage retention than C3 but higher coverage-bounded lifetime at every evaluated threshold. At θ=0.7, C3 had longer average coverage retention than EARDC with $R_{\mathrm{CH}}=R_{\mathrm{comm}}$ (4224 versus 829 rounds). EARDC had a longer average coverage-bounded lifetime (7884 versus 4750 rounds). Scheduled reclustering allowed EARDC coverage to rise above the threshold after coverage first fell below it. These later qualifying rounds contributed to coverage-bounded lifetime. The sensitivity analysis showed that $R_{\mathrm{CH}}$ affected network lifetime, coverage, and active participation differently at each deployment density.

The ERD-only ablation showed that EARDC membership decisions and the resulting sleep states contributed jointly to the lower active ratio and longer network lifetime beyond residual-energy-aware RD CH selection alone. The ablation did not separate the individual contributions of membership decisions and sleep-state control because the sleep state followed the membership decision. At λ=0.001, ERD-only provided higher coverage retention and coverage-bounded lifetime from θ=0.9 to 0.6. At λ=0.005 and 0.02, EARDC provided higher values for both coverage measures at every evaluated threshold. The coverage effect therefore depends on deployment density and the selected threshold. The omnibus tests identified protocol-level differences, and the $\eta^{2}$ values and pairwise mean differences with 95% confidence intervals quantified their magnitude. EARDC’s principal advantage is longer network lifetime with selective sensing participation, accompanied by tradeoffs in coverage retention and redundancy.

All protocols used direct CH-to-base-station transmission. Protocol-specific routing differences were therefore excluded from the evaluation. The simulation energy model included the local messages required for the reaction–diffusion update, CH selection, and membership control. The topology evaluation considered connectivity changes caused by node energy depletion under fixed sensor positions and communication radii. The performance results are specific to the three homogeneous PPP densities, centered base station, square monitoring field, and fixed sensing and communication radii used in the simulations.

Future simulation studies should evaluate multi-hop routing, mobile sinks, nonuniform sensor deployment, heterogeneous sensing and communication ranges, and different base-station locations. Node mobility, time-varying links, and abrupt node failures also require separate evaluation. Additional sensitivity studies should examine $T_{\mathrm{epoch}}$ and the reaction–diffusion parameters.

Analytical work can develop CH and active-node models that do not require simulated CH density or an estimated non-CH participation probability. Models that account for dependence among active-node locations may improve the coverage approximation. Separate membership-only and sleep-only variants would require explicit activity rules for non-CH nodes that are not accepted as members before the individual effects can be evaluated. Comparative studies should include recent distributed coverage-control and joint clustering-and-coverage protocols under common deployment, routing, and energy assumptions. Analysis under nonuniform residual energy should also examine convergence and stability.

## Figures and Tables

**Table 1:** Summary of the WSN coverage and clustering methods reviewed in this section.

Category	Methods	Primary Mechanism	Main Limitation
Conventional clustering	LEACH [7]	Probabilistic CH rotation	CH selection does not consider residual energy, area coverage, or sensing redundancy
Threshold-based reporting	TEEN and APTEEN [25,26]	Hard and soft thresholds control data reporting	Controls reporting events rather than active sensing participation for area coverage
Energy-aware clustering	HEED and variants [8,23,24]	Distributed CH election based on residual energy and intra-cluster communication cost	Does not control active sensing participation for area coverage
Coverage-aware clustering	C3 [28]	RSSI-derived virtual rings, clustering, and redundancy-based sleep scheduling	Virtual ring reconfiguration is initiated by a sink or reference node
Learning-based coverage	DRL and GNN methods [15,17]	Learning-based active sensor configuration and coverage-hole detection	May require training data, network-wide state, or node-location information, depending on the method
Population-based metaheuristics	PSO, ACO, ABC, GA, DE, ICS-MS, SHOE, HEHO-CA-OCHS [18,32,33,35,37,39,40,41]	Population-based optimization of CH selection, routing, cluster formation, or sensor placement	Iterative optimization can add computation and coordination overhead
Reaction–diffusion	RD clustering models [42,47]	Distributed CH selection and topology formation through local activator–inhibitor updates	Prior RD methods do not integrate residual energy into both CH selection and active sensing control

**Table 2:** Per-sensor state maintained by EARDC.

State Variable	Symbol or Value	Role in EARDC
Activator value	$a_{i}\left(t\right)$	Local activator value used in the energy-aware update and the local-maximum comparison for CH selection.
Inhibitor value	$h_{i}\left(t\right)$	Local inhibitor value used in the energy-aware update that determines the activator values evaluated during CH selection.
Residual energy	$E_{i}\left(t\right),{\hat{E}}_{i}\left(t\right)$	Residual and normalized residual energy of node *i* at round *t*.
Cluster state	CH flag, selected CH identifier, accepted-member status	Current clustering status used for CH duties and member participation.
Sleep state	Sleep flag and timer $\tau_{i}\left(t\right)$	Temporary sleep state. A value $\tau_{i}\left(t\right)>0$ disables sensing and communication until the next scheduled clustering epoch; $\tau_{i}\left(t\right)=0$ indicates that the node is not sleeping.
Local range tests	Local distance estimates and radius checks	Local range information used for CH selection, cluster joining, and sleep-state control.

**Table 3:** Coverage and performance metrics evaluated from the active sensing set.

Metric	Definition or Description
Instantaneous Coverage $R_{c}\left(t\right)$	$\frac{\left|\{g\in G:n_{g}\left(t\right)>0\}\right|}{\left|G\right|}$: grid-cell coverage from A(t)
Average Coverage ${\bar{R}}_{c}$	$\frac{1}{T}\sum_{t=1}^{T}R_{c}\left(t\right)$: average coverage over recorded rounds
Overlap Ratio $R_{\mathrm{overlap}}\left(t\right)$	$\frac{\left|\{g\in G_{\mathrm{cov}}\left(t\right):n_{g}\left(t\right)>1\}\right|}{|G_{\mathrm{cov}}\left(t\right)|}$
Redundancy Degree $D_{\mathrm{red}}\left(t\right)$	Average value of $n_{g}\left(t\right)-1$ over covered grid cells
Redundant Node Ratio $R_{\mathrm{node}}\left(t\right)$	$\frac{N_{\mathrm{red}}\left(t\right)}{\left|A(t)\right|}$: fraction of active nodes whose cells are fully redundant
Network Lifetime $T_{\mathrm{life}}$	$\max\{t:N_{\mathrm{alive}}\left(t\right)>0\}$
First Node Death $T_{\mathrm{FND}}$	$\min\{t:N_{\mathrm{alive}}\left(t\right)<N_{\mathrm{initial}}\}$
Coverage Retention $T_{\mathrm{cov}}\left(\theta \right)$	First round with $R_{c}\left(t\right)<\theta$
Coverage-Bounded Lifetime $L_{\mathrm{cov}}\left(\theta \right)$	Number of rounds with $R_{c}\left(t\right)\geq \theta$
Cluster Stability $S_{\mathrm{cluster}}$	std(|H(t)|): temporal variation in the number of clusters

**Table 4:** Parameters used in the hexagonal reference analysis.

Symbol	Description	Expression or Range
$L_{x}$	Length of region	–
$L_{y}$	Width of region	–
$|A_{\mathrm{total}}|$	Area of monitoring region	$L_{x}L_{y}$
$\lambda_{c}$	Center-to-center distance between neighboring CHs	–
$|A_{\mathrm{hex}}|$	Area of hexagonal cell (per cluster)	$\frac{\sqrt{3}}{2}\lambda_{c}^{2}$
$N_{s}$	Number of clusters	$\frac{2L_{x}L_{y}}{\sqrt{3}\lambda_{c}^{2}}$
$d_{s}$	Diameter of the idealized circular sensing footprint	$\lambda_{c}\leq d_{s}\leq \frac{2}{\sqrt{3}}\lambda_{c}$
$|A_{s}|$	Area covered by a single cluster	$\frac{\pi d_{s}^{2}}{4}$
$\Gamma (d_{s})$	Aggregate sensing-area load	$\frac{N_{s}\left|A_{s}\right|}{|A_{\mathrm{total}}|}\approx \frac{\pi d_{s}^{2}}{2\sqrt{3}\lambda_{c}^{2}}$

**Table 5:** Simulation parameters used for the clustering and coverage evaluation.

No.	Parameter	Value
1	Deployment density, λ	0.001, 0.005, 0.02 nodes/$m^{2}$
2	Monitoring area	100m×100m
3	Deployment model	Homogeneous PPP
4	Mobility model	Static nodes
5	Base-station location	(50,50)m
6	Initial energy per node	1J
7	Communication radius, $R_{\mathrm{comm}}$	35m
8	Sensing radius, $R_{s}$	25m
9	Data packet size	4096 bits (512 bytes)
10	Control message size	128 bits
11	Activator broadcast size	48 bits
12	Activator and inhibitor exchange size	80 bits
13	Sensing energy	50nJ/bit
14	Transmitter and receiver electronics energy, $E_{\mathrm{elec}}$	50nJ/bit
15	Aggregation energy at CH	5nJ/bit
16	Transmit amplifier energy, $\epsilon_{\mathrm{fs}}$	$10\;\mathrm{pJ}/\mathrm{bit}/m^{2}$
17	Number of independent runs	300
EARDC parameters		
18	CH separation radius	$R_{s}$, $1.2R_{s}$, $R_{\mathrm{comm}}$
19	Clustering-epoch period, $T_{\mathrm{epoch}}$	20 rounds
20	Inner joining distance	$0.8R_{s}$
21	Autocatalytic coefficient, σ	1.0
22	Activator decay rate, $\mu_{a}$	0.1
23	Inhibitor decay rate, $\mu_{h}$	0.2
24	Activator basal production rate, $\rho_{a}$	0.04
25	Inhibitor basal production rate, $\rho_{h}$	0.02
26	Activator diffusion coefficient, $D_{a}$	0.005
27	Inhibitor diffusion coefficient, $D_{h}$	0.2
28	Energy-aware update coefficient, α	0.01
29	Inhibitor denominator safeguard, $\epsilon_{h}$	$10^{-6}$
Baseline protocol parameters		
30	LEACH CH probability, *p*	0.05
31	HEED initial CH probability, $C_{\mathrm{prob}}$	0.05
32	HEED minimum CH probability, $C_{\min}$	0.001
33	HEED maximum iterations	10
34	C3 gateway forwarding	Disabled for clustering-only comparison
35	WIFN optimal CH probability	0.05
36	WIFN population size and iterations	20 and 10

**Table 6:** Lifetime-averaged metric summary (mean±std) for deployment density λ=0.001 nodes/$m^{2}$. Coverage, overlap, redundancy, and active ratio are evaluated from the active sensing set.

Protocol	Lifetime	FND	Alive	Alive Ratio	Active Ratio	Clusters	Coverage	Overlap Ratio	Redundancy Degree	Redundant Node Ratio	Cluster Stability
C3	5099 ± 1444	1613 ± 343	6.688 ± 2.242	0.663 ± 0.247	0.306 ± 0.213	2.402 ± 1.290	0.382 ± 0.193	0.098 ± 0.094	0.107 ± 0.104	0.004 ± 0.006	0.656 ± 0.381
EARDC ($R_{\mathrm{CH}}\geq R_{s}$)	5547 ± 1646	1840 ± 156	5.961 ± 3.320	0.571 ± 0.340	0.364 ± 0.232	3.014 ± 1.774	0.451 ± 0.229	0.175 ± 0.101	0.192 ± 0.116	0.003 ± 0.003	0.706 ± 0.476
EARDC ($R_{\mathrm{CH}}\geq 1.2\;R_{s}$)	5552 ± 1632	1819 ± 185	5.957 ± 3.042	0.586 ± 0.330	0.359 ± 0.225	2.514 ± 1.323	0.431 ± 0.210	0.168 ± 0.092	0.187 ± 0.109	0.003 ± 0.003	0.571 ± 0.342
EARDC ($R_{\mathrm{CH}}\geq R_{\mathrm{comm}}$)	5440 ± 1605	1805 ± 272	5.904 ± 2.947	0.598 ± 0.327	0.363 ± 0.220	2.264 ± 1.098	0.424 ± 0.203	0.168 ± 0.091	0.187 ± 0.108	0.003 ± 0.003	0.491 ± 0.254
HEED	3222 ± 636	1775 ± 190	6.736 ± 3.972	0.681 ± 0.382	0.681 ± 0.382	3.547 ± 1.551	0.577 ± 0.264	0.364 ± 0.259	0.596 ± 0.450	0.075 ± 0.064	0.999 ± 0.127
LEACH	1571 ± 70	1356 ± 111	8.987 ± 1.959	0.928 ± 0.190	0.929 ± 0.189	2.098 ± 1.828	0.718 ± 0.124	0.538 ± 0.100	0.907 ± 0.184	0.120 ± 0.032	2.005 ± 0.248
WIFN	3672 ± 808	2422 ± 307	7.849 ± 3.108	0.793 ± 0.327	0.793 ± 0.327	1.051 ± 0.249	0.635 ± 0.217	0.470 ± 0.170	0.803 ± 0.310	0.108 ± 0.048	0.294 ± 0.054

**Table 7:** Lifetime-averaged metric summary (mean±std) for deployment density λ=0.005 nodes/$m^{2}$. Coverage, overlap, redundancy, and active ratio are evaluated from the active sensing set.

Protocol	Lifetime	FND	Alive	Alive Ratio	Active Ratio	Clusters	Coverage	Overlap Ratio	Redundancy Degree	Redundant Node Ratio	Cluster Stability
C3	8320 ± 319	1907 ± 471	35.780 ± 14.750	0.708 ± 0.293	0.110 ± 0.033	4.986 ± 1.453	0.699 ± 0.166	0.144 ± 0.043	0.154 ± 0.046	0.009 ± 0.006	0.762 ± 0.326
EARDC ($R_{\mathrm{CH}}\geq R_{s}$)	11,419 ± 1829	1504 ± 250	28.468 ± 16.040	0.563 ± 0.322	0.164 ± 0.105	3.881 ± 1.817	0.664 ± 0.275	0.434 ± 0.230	0.734 ± 0.517	0.112 ± 0.122	1.144 ± 0.301
EARDC ($R_{\mathrm{CH}}\geq 1.2\;R_{s}$)	11,159 ± 1775	1459 ± 275	29.377 ± 15.578	0.584 ± 0.316	0.163 ± 0.104	3.222 ± 1.338	0.643 ± 0.258	0.439 ± 0.226	0.766 ± 0.533	0.120 ± 0.127	0.998 ± 0.223
EARDC ($R_{\mathrm{CH}}\geq R_{\mathrm{comm}}$)	11,258 ± 1731	1447 ± 328	30.547 ± 15.121	0.606 ± 0.306	0.159 ± 0.098	2.800 ± 1.025	0.620 ± 0.236	0.446 ± 0.220	0.785 ± 0.527	0.118 ± 0.121	0.933 ± 0.171
HEED	2247 ± 120	1020 ± 186	38.977 ± 16.990	0.777 ± 0.338	0.777 ± 0.338	9.379 ± 3.546	0.905 ± 0.226	0.864 ± 0.277	5.283 ± 2.406	0.766 ± 0.331	6.769 ± 1.021
LEACH	1456 ± 37	1237 ± 33	45.477 ± 11.416	0.921 ± 0.230	0.921 ± 0.229	2.367 ± 0.482	0.959 ± 0.151	0.940 ± 0.178	6.150 ± 1.647	0.878 ± 0.218	1.346 ± 0.083
WIFN	5478 ± 492	2758 ± 106	30.338 ± 21.845	0.606 ± 0.436	0.606 ± 0.436	1.774 ± 0.623	0.769 ± 0.295	0.714 ± 0.331	4.087 ± 3.073	0.569 ± 0.428	0.724 ± 0.064

**Table 8:** Lifetime-averaged metric summary (mean±std) for deployment density λ=0.02 nodes/$m^{2}$. Coverage, overlap, redundancy, and active ratio are evaluated from the active sensing set.

Protocol	Lifetime	FND	Alive	Alive Ratio	Active Ratio	Clusters	Coverage	Overlap Ratio	Redundancy Degree	Redundant Node Ratio	Cluster Stability
C3	6566 ± 408	1300 ± 158	126.501 ± 74.512	0.635 ± 0.372	0.033 ± 0.010	6.273 ± 2.007	0.729 ± 0.246	0.143 ± 0.054	0.154 ± 0.059	0.015 ± 0.007	0.750 ± 0.317
EARDC ($R_{\mathrm{CH}}\geq R_{s}$)	16,094 ± 2250	1599 ± 383	107.642 ± 68.002	0.540 ± 0.342	0.101 ± 0.075	3.817 ± 1.327	0.721 ± 0.269	0.634 ± 0.288	2.749 ± 2.306	0.413 ± 0.316	1.254 ± 0.158
EARDC ($R_{\mathrm{CH}}\geq 1.2\;R_{s}$)	15,712 ± 2248	1705 ± 430	117.988 ± 64.977	0.591 ± 0.327	0.101 ± 0.067	2.842 ± 0.772	0.679 ± 0.219	0.674 ± 0.272	3.098 ± 2.232	0.438 ± 0.289	1.070 ± 0.161
EARDC ($R_{\mathrm{CH}}\geq R_{\mathrm{comm}}$)	16,327 ± 2308	1784 ± 418	128.435 ± 61.019	0.639 ± 0.305	0.096 ± 0.059	2.297 ± 0.537	0.635 ± 0.180	0.699 ± 0.262	3.250 ± 2.119	0.441 ± 0.266	0.954 ± 0.158
HEED	2412 ± 53	394 ± 125	144.358 ± 66.061	0.724 ± 0.332	0.724 ± 0.332	39.662 ± 28.713	0.953 ± 0.152	0.941 ± 0.177	21.998 ± 9.972	0.901 ± 0.249	50.323 ± 8.037
LEACH	1549 ± 25	1176 ± 20	173.602 ± 57.908	0.870 ± 0.290	0.870 ± 0.289	8.780 ± 2.682	0.949 ± 0.176	0.956 ± 0.163	25.997 ± 9.282	0.933 ± 0.211	3.108 ± 0.188
WIFN	5231 ± 254	2882 ± 51	128.063 ± 86.503	0.642 ± 0.433	0.642 ± 0.433	2.424 ± 0.720	0.887 ± 0.213	0.898 ± 0.183	19.458 ± 13.181	0.807 ± 0.308	1.174 ± 0.106

**Table 9:** Coverage retention $T_{\mathrm{cov}}\left(\theta \right)$ for the seven evaluated configurations (rounds, mean±std).

Density λ (Nodes/$m^{2}$)	Configuration	θ=0.9	θ=0.8	θ=0.7	θ=0.6	θ=0.5
0.001	C3	19±150	149±425	527±728	1000±845	1503±729
	EARDC ($R_{\mathrm{CH}}\geq R_{s}$)	37±243	367±729	961±973	1514±968	2074±896
	EARDC ($R_{\mathrm{CH}}\geq 1.2\;R_{s}$)	1±7	110±380	603±850	1208±1004	1741±1006
	EARDC ($R_{\mathrm{CH}}\geq R_{\mathrm{comm}}$)	1±8	81±327	368±718	797±963	1424±1034
	HEED	279±665	970±960	1513±841	1892±558	2108±435
	LEACH	196±480	633±692	1031±630	1249±494	1389±326
	WIFN	412±945	1147±1312	1967±1216	2459±922	2802±527
0.005	C3	91±287	815±815	2634±1460	5011±1401	6384±924
	EARDC ($R_{\mathrm{CH}}\geq R_{s}$)	1462±1048	2951±1155	4016±1272	5179±1588	6602±1634
	EARDC ($R_{\mathrm{CH}}\geq 1.2\;R_{s}$)	785±889	1900±1163	2888±1353	3825±1551	4929±1897
	EARDC ($R_{\mathrm{CH}}\geq R_{\mathrm{comm}}$)	341±621	1129±1073	2009±1289	2766±1420	3823±1850
	HEED	1880±52	1939±48	1977±45	2015±48	2047±47
	LEACH	1366±25	1380±25	1389±25	1396±25	1402±25
	WIFN	3224±204	3421±183	3585±266	3848±425	4177±490
0.02	C3	230±338	1678±990	4224±650	4774±337	5125±307
	EARDC ($R_{\mathrm{CH}}\geq R_{s}$)	752±824	1577±1558	2976±2950	4679±3921	6862±4528
	EARDC ($R_{\mathrm{CH}}\geq 1.2\;R_{s}$)	356±414	783±739	1258±1008	1822±1509	2844±2414
	EARDC ($R_{\mathrm{CH}}\geq R_{\mathrm{comm}}$)	177±228	416±466	829±764	1226±982	1699±1274
	HEED	2179±55	2237±49	2273±45	2298±43	2322±42
	LEACH	1394±15	1433±13	1457±13	1470±13	1481±13
	WIFN	3945±332	4183±334	4382±281	4578±222	4746±211

**Table 10:** Coverage-bounded lifetime $L_{\mathrm{cov}}\left(\theta \right)$ for the seven evaluated configurations (rounds, mean±std).

Density λ (Nodes/$m^{2}$)	Configuration	θ=0.9	θ=0.8	θ=0.7	θ=0.6	θ=0.5
0.001	C3	25±159	195±457	632±748	1194±832	1765±778
	EARDC ($R_{\mathrm{CH}}\geq R_{s}$)	131±427	668±847	1309±966	1896±901	2521±896
	EARDC ($R_{\mathrm{CH}}\geq 1.2\;R_{s}$)	48±222	434±688	1075±942	1753±995	2347±1009
	EARDC ($R_{\mathrm{CH}}\geq R_{\mathrm{comm}}$)	57±234	401±660	959±904	1605±1010	2230±978
	HEED	279±665	970±960	1513±841	1892±558	2108±435
	LEACH	196±480	633±692	1031±630	1249±494	1389±326
	WIFN	412±945	1147±1312	1967±1216	2459±922	2802±527
0.005	C3	495±504	2882±1094	5302±956	6566±639	7188±483
	EARDC ($R_{\mathrm{CH}}\geq R_{s}$)	3460±865	5116±933	6445±995	7443±1064	8205±1087
	EARDC ($R_{\mathrm{CH}}\geq 1.2\;R_{s}$)	2748±871	4356±965	5801±1017	6950±1033	7908±1066
	EARDC ($R_{\mathrm{CH}}\geq R_{\mathrm{comm}}$)	2188±812	3766±964	5297±1063	6642±1077	7723±1125
	HEED	1880±52	1939±48	1977±45	2015±48	2047±47
	LEACH	1366±25	1380±25	1389±25	1396±25	1403±25
	WIFN	3224±204	3421±183	3585±266	3848±425	4177±490
0.02	C3	1627±482	4132±392	4750±295	5084±288	5352±286
	EARDC ($R_{\mathrm{CH}}\geq R_{s}$)	6682±1470	8997±1300	10,507±1219	11,628±1224	12,523±1271
	EARDC ($R_{\mathrm{CH}}\geq 1.2\;R_{s}$)	4098±1330	6904±1261	9032±1108	10,780±1113	12,077±1165
	EARDC ($R_{\mathrm{CH}}\geq R_{\mathrm{comm}}$)	2668±1008	5487±1245	7884±1195	10,121±1088	11,830±1115
	HEED	2179±55	2237±49	2273±45	2298±43	2322±42
	LEACH	1394±15	1433±13	1457±13	1470±13	1481±13
	WIFN	3945±332	4183±334	4382±281	4578±222	4746±211

**Table 11:** Comparison of the Matérn Type II hard-core reference with simulated CH density and of the conditional active-node density estimate with simulation. The active-node density estimate uses the simulated CH density and ${\hat{q}}_{\mathrm{MS}}$ estimated from an independent set of runs. Bracketed ranges following $\lambda_{\mathrm{CH}}^{\mathrm{sim}}$ and $\lambda_{\mathrm{act}}^{\mathrm{sim}}$ are 95% confidence intervals for the means from 150 validation runs. The column $|e_{\mathrm{MII}}|$ gives the absolute percentage difference from the Matérn reference. The column $|e_{\mathrm{act}}|$ gives the absolute percentage error relative to the conditional active-node density estimate.

(a) CH-Density Comparison				
λ	$R_{\mathrm{CH}}$	$\lambda_{\mathrm{MII}}$	$\lambda_{\mathrm{CH}}^{\mathrm{sim}}$	$|e_{\mathrm{MII}}|$
0.001	$R_{s}$	0.000438	0.000533 [0.000513, 0.000554]	21.8%
0.001	$1.2R_{s}$	0.000333	0.000423 [0.000405, 0.000442]	27.2%
0.001	$R_{\mathrm{comm}}$	0.000254	0.000355 [0.000340, 0.000371]	39.7%
0.005	$R_{s}$	0.000509	0.000673 [0.000656, 0.000690]	32.1%
0.005	$1.2R_{s}$	0.000354	0.000537 [0.000522, 0.000553]	51.9%
0.005	$R_{\mathrm{comm}}$	0.000260	0.000448 [0.000433, 0.000462]	72.3%
0.02	$R_{s}$	0.000509	0.000450 [0.000433, 0.000467]	11.6%
0.02	$1.2R_{s}$	0.000354	0.000327 [0.000314, 0.000340]	7.5%
0.02	$R_{\mathrm{comm}}$	0.000260	0.000254 [0.000245, 0.000263]	2.3%
**(b) Active-Node Density Comparison**				
λ	$R_{\mathrm{CH}}$	$\lambda_{\mathrm{act}}^{\mathrm{cond}}$	$\lambda_{\mathrm{act}}^{\mathrm{sim}}$	$|e_{\mathrm{act}}|$
0.001	$R_{s}$	0.000656	0.000648 [0.000623, 0.000674]	1.2%
0.001	$1.2R_{s}$	0.000629	0.000607 [0.000581, 0.000632]	3.6%
0.001	$R_{\mathrm{comm}}$	0.000617	0.000578 [0.000553, 0.000603]	6.3%
0.005	$R_{s}$	0.001690	0.001654 [0.001616, 0.001692]	2.1%
0.005	$1.2R_{s}$	0.001686	0.001671 [0.001636, 0.001707]	0.9%
0.005	$R_{\mathrm{comm}}$	0.001647	0.001657 [0.001620, 0.001695]	0.6%
0.02	$R_{s}$	0.004492	0.004523 [0.004422, 0.004624]	0.7%
0.02	$1.2R_{s}$	0.004137	0.004090 [0.003974, 0.004206]	1.1%
0.02	$R_{\mathrm{comm}}$	0.003738	0.003583 [0.003481, 0.003685]	4.1%

**Table 12:** Estimated conditional non-CH participation probability ${\hat{q}}_{\mathrm{MS}}$ for each deployment density and CH separation setting. The estimate is the unweighted mean over 150 estimation runs. The bootstrap 95% confidence interval is bounded by the 2.5th and 97.5th percentiles obtained from 2000 resamples, with replacement, of the estimates from the 150 estimation runs. The remaining 150 runs are reserved for validation.

λ	$R_{\mathrm{CH}}$	${\hat{q}}_{\mathrm{MS}}$	Bootstrap 95% CI
0.001	$R_{s}$	0.263	[0.232, 0.295]
0.001	$1.2R_{s}$	0.357	[0.329, 0.384]
0.001	$R_{\mathrm{comm}}$	0.406	[0.379, 0.435]
0.005	$R_{s}$	0.235	[0.228, 0.242]
0.005	$1.2R_{s}$	0.257	[0.249, 0.266]
0.005	$R_{\mathrm{comm}}$	0.263	[0.255, 0.271]
0.02	$R_{s}$	0.207	[0.202, 0.211]
0.02	$1.2R_{s}$	0.194	[0.188, 0.199]
0.02	$R_{\mathrm{comm}}$	0.176	[0.171, 0.182]

**Table 13:** Comparison of conditional Poisson coverage estimates and simulated area coverage. The conditional estimate $R_{c}^{\mathrm{cond}}$ uses the active-node density in Table 11 and accounts for sensing disks truncated by the deployment boundary. The simulation confidence interval is the 95% confidence interval for the mean $R_{c}^{\mathrm{sim}}$ from 150 validation runs. The column $|e_{c}|$ gives the absolute percentage error relative to $R_{c}^{\mathrm{cond}}$.

λ	$R_{\mathrm{CH}}$	$R_{c}^{\mathrm{cond}}$	$R_{c}^{\mathrm{sim}}$	Simulation CI	$|e_{c}|$
0.001	$R_{s}$	0.631	0.725	[0.706, 0.745]	15.0%
0.001	$1.2R_{s}$	0.616	0.685	[0.663, 0.706]	11.2%
0.001	$R_{\mathrm{comm}}$	0.609	0.667	[0.645, 0.688]	9.5%
0.005	$R_{s}$	0.912	0.974	[0.971, 0.978]	6.9%
0.005	$1.2R_{s}$	0.911	0.955	[0.949, 0.962]	4.9%
0.005	$R_{\mathrm{comm}}$	0.907	0.931	[0.922, 0.940]	2.7%
0.02	$R_{s}$	0.996	0.921	[0.909, 0.933]	7.6%
0.02	$1.2R_{s}$	0.995	0.850	[0.834, 0.866]	14.5%
0.02	$R_{\mathrm{comm}}$	0.992	0.772	[0.755, 0.789]	22.1%

**Table 14:** Ranges of Holm-adjusted *p*-values for pairwise comparisons between EARDC with $R_{\mathrm{CH}}=R_{\mathrm{comm}}$ and C3, HEED, LEACH, and WIFN.

Density λ (Nodes/$m^{2}$)	Network Lifetime	Active Ratio	Coverage
0.001	0.021–0.032	0.021	0.021
0.005	0.021	0.021	0.021
0.02	0.021	0.021	0.021

**Table 15:** Pairwise differences in run-level averages between EARDC with $R_{\mathrm{CH}}=R_{\mathrm{comm}}$ and the clustering baselines. In each entry, the value before the brackets is the difference $\Delta ={\bar{x}}_{\mathrm{EARDC}}-{\bar{x}}_{\mathrm{baseline}}$, and the bracketed range is its 95% confidence interval. A positive Δ means that EARDC has a higher average for the specified metric, and a negative Δ means that EARDC has a lower average.

λ (Nodes/$m^{2}$)	Baseline	Lifetime (Rounds)	Active Ratio	Coverage
0.001	C3	341[96,586]	0.046[0.019,0.074]	0.042[0.026,0.057]
	HEED	2218[2021,2414]	−0.294[−0.313,−0.274]	−0.147[−0.164,−0.131]
	LEACH	3869[3686,4051]	−0.520[−0.536,−0.504]	−0.267[−0.286,−0.248]
	WIFN	1767[1563,1972]	−0.422[−0.442,−0.401]	−0.209[−0.225,−0.193]
0.005	C3	2938[2738,3138]	0.054[0.051,0.057]	−0.061[−0.069,−0.053]
	HEED	9011[8814,9208]	−0.615[−0.620,−0.610]	−0.270[−0.278,−0.261]
	LEACH	9802[9605,9998]	−0.758[−0.762,−0.755]	−0.321[−0.328,−0.313]
	WIFN	5779[5575,5984]	−0.448[−0.454,−0.441]	−0.140[−0.150,−0.130]
0.02	C3	9762 [9495, 10,028]	0.066[0.064,0.067]	−0.076[−0.084,−0.068]
	HEED	13,915 [13,653, 14,177]	−0.625[−0.628,−0.623]	−0.298[−0.304,−0.291]
	LEACH	14,778 [14,516, 15,040]	−0.772[−0.774,−0.770]	−0.293[−0.300,−0.286]
	WIFN	11,096 [10,833, 11,360]	−0.544[−0.548,−0.541]	−0.237[−0.244,−0.229]

**Table 16:** Effect of membership and sensing participation control on network lifetime, FND, and active ratio (mean±std). EARDC and ERD-only use $R_{\mathrm{CH}}=R_{\mathrm{comm}}$ at all deployment densities.

Density λ (Nodes/$m^{2}$)	Configuration	Network Lifetime	FND	Active Ratio
0.001	EARDC	5440±1605	1805±272	0.363±0.221
	ERD-only	2668±434	1041±226	0.650±0.307
0.005	EARDC	11,258 ±1731	1447±328	0.159±0.098
	ERD-only	4395±721	488±73	0.352±0.242
0.02	EARDC	16,327 ±2308	1784±418	0.096±0.059
	ERD-only	7469±711	123±60	0.198±0.131

**Table 17:** Coverage retention and coverage-bounded lifetime for EARDC and ERD-only with $R_{\mathrm{CH}}=R_{\mathrm{comm}}$ (rounds, mean±std).

Density	Configuration	Metric	θ=0.9	θ=0.8	θ=0.7	θ=0.6	θ=0.5
0.001	EARDC	$T_{\mathrm{cov}}$	0.9±8.3	80.5±327.3	367.7±718.4	797.3±963.4	1424.1±1033.6
		$L_{\mathrm{cov}}$	57.0±234.3	401.2±660.1	958.9±903.9	1604.9±1009.6	2229.9±977.5
	ERD-only	$T_{\mathrm{cov}}$	22.6±139.0	192.1±386.1	508.0±545.2	819.4±574.5	1170.8±562.8
		$L_{\mathrm{cov}}$	109.1±307.1	592.0±665.1	1138.1±753.0	1620.3±617.6	1953.6±438.2
0.005	EARDC	$T_{\mathrm{cov}}$	340.5±620.6	1129.3±1073.2	2009.0±1289.2	2765.9±1419.6	3823.3±1850.0
		$L_{\mathrm{cov}}$	2188.4±812.1	3766.2±964.1	5297.2±1062.7	6641.7±1077.1	7723.2±1125.3
	ERD-only	$T_{\mathrm{cov}}$	219.3±245.4	443.5±286.8	619.1±337.6	801.6±392.9	1044.2±481.9
		$L_{\mathrm{cov}}$	1102.1±419.5	1678.2±424.3	2220.1±359.3	2697.7±313.0	3071.9±344.0
0.02	EARDC	$T_{\mathrm{cov}}$	176.6±227.7	416.1±465.6	829.2±764.0	1226.4±981.9	1698.6±1273.6
		$L_{\mathrm{cov}}$	2668.1±1007.9	5486.6±1244.9	7883.6±1194.6	10120.6±1087.6	11829.8±1114.7
	ERD-only	$T_{\mathrm{cov}}$	109.5±87.4	196.3±107.3	248.2±108.3	290.4±110.3	336.4±127.7
		$L_{\mathrm{cov}}$	549.5±349.7	1266.5±579.2	2163.2±559.9	3050.7±368.3	3969.0±287.0

## Data Availability

The data presented in this study are available on request from the corresponding author.
